# Design, Synthesis, and Biological Evaluation of Marine Lissodendrins B Analogues as Modulators of ABCB1-Mediated Multidrug Resistance

**DOI:** 10.3390/md21050314

**Published:** 2023-05-20

**Authors:** Chaoming Wang, Jinman Zhang, Xianfeng Wei, Mengke Yang, Weiping Ma, Rilei Yu, Ming Liu, Tao Jiang

**Affiliations:** 1 Key Laboratory of Marine Drugs, The Ministry of Education of China, School of Medicine and Pharmacy, Ocean University of China, Qingdao 266003, China; wangchaoming315@163.com (C.W.); hdszhjm@126.com (J.Z.); weixfeng427@163.com (X.W.); yangmengke@stu.ouc.edu.cn (M.Y.); maweiping1990@163.com (W.M.); jiangtao@ouc.edu.cn (T.J.); 2 Innovation Center for Marine Drug Screening & Evaluation and Laboratory for Marine Drugs and Bioproducts, Qingdao National Laboratory for Marine Science and Technology, Qingdao 266003, China

**Keywords:** Lissodendrins B analogues, Y-shaped scaffold, tetrahydroisoquinoline, multidrug resistance, ABCB1 inhibitors

## Abstract

Multidrug resistance (MDR) caused by ATP-Binding Cassette Subfamily B Member 1 (ABCB1, P-glycoprotein, P-gp) is a major barrier for the success of chemotherapy in clinics. In this study, we designed and synthesized a total of 19 Lissodendrins B analogues and tested their ABCB1-mediated MDR reversal activity in doxorubicin (DOX)-resistant K562/ADR and MCF-7/ADR cells. Among all derivatives, compounds **D_1_**, **D_2_**, and **D_4_** with a dimethoxy-substituted tetrahydroisoquinoline fragment possessed potent synergistic effects with DOX and reversed ABCB1-mediated drug resistance. Notably, the most potent compound **D_1_** merits multiple activities, including low cytotoxicity, the strongest synergistic effect, and effectively reversing ABCB1-mediated drug resistance of K562/ADR (RF = 1845.76) and MCF-7/ADR cells (RF = 207.86) to DOX. As a reference substance, compound **D_1_** allows for additional mechanistic studies on ABCB1 inhibition. The synergistic mechanisms were mainly related to the increased intracellular accumulation of DOX via inhibiting the efflux function of ABCB1 rather than from affecting the expression level of ABCB1. These studies suggest that compound **D_1_** and its derivatives might be potential MDR reversal agents acting as ABCB1 inhibitors in clinical therapeutics and provide insight into a design strategy for the development of ABCB1 inhibitors.

## 1. Introduction

Cancers are common and frequently occurring diseases that seriously threaten human health, and chemotherapy is still the most effective method for treating cancer [1]. However, the occurrence of termed multidrug resistance (MDR) in cancer cells has led to chemotherapy being greatly ineffective [2,3]. The prime mechanism may be that increased efflux of multitudinous chemotherapeutic agents causes chemotherapy failure due to decreased intracellular drug levels and, thus, limited efficacy. Overexpression of the drug efflux ABCB1 transporter is considered to be the most pivotal reason for MDR [4,5]. Obviously, ABCB1 is an ideal and predominant target for reversing MDR by inhibiting its efflux function.

**Figure 1:**
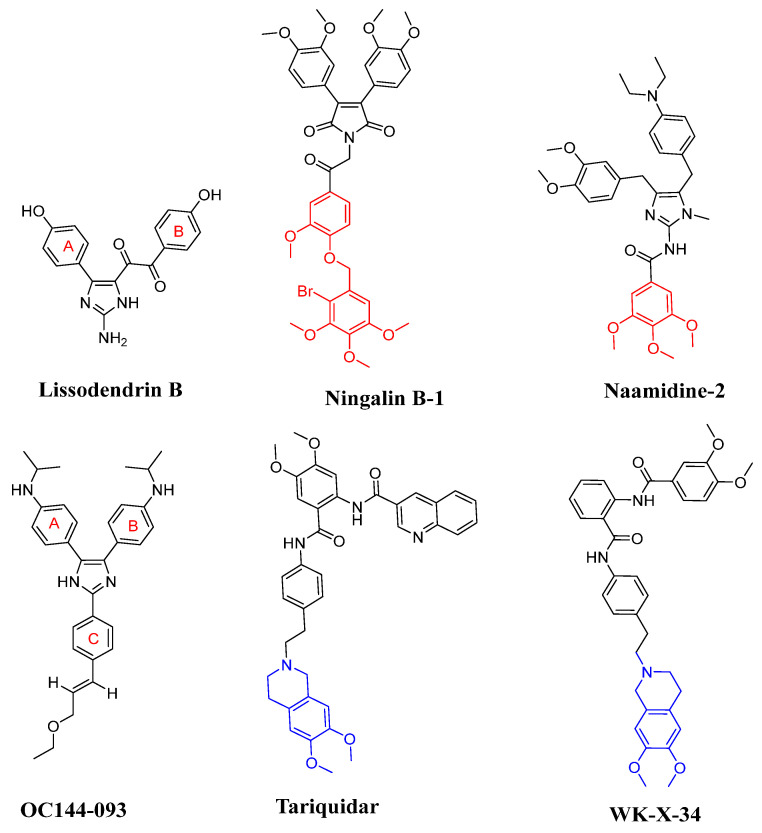
Representative marine products (Lissodendrins B, Ningalin B-1 and Naamidine-1) and third-generation ABCB1 inhibitors (OC144-093, tariquidar, and WK-X-34).

In recent years, many derivatives of natural products have been successfully reported as high-efficiency ABCB1 reversal agents, such as methylated quercetin analogues [6], methylated Ningalin B analogues [7,8], curcumin analogues [9], and methylated Naamidines analogues [10]. These compounds could effectively reverse the ABCB1-mediated tumor MDR and show low cytotoxicity. Among them, the marine alkaloids Ningalin B-1 and Naamidine-1 have preferable ABCB1 regulatory activity and reversed the tumor MDR (Figure 1). Both Ningalin B-1 and Naamidine-1 with three methoxy groups of C ring at 1 μM can sensitize LCC6MDR cells toward paclitaxel by 48- and 26.4-fold, respectively, and have non-toxic side effects on LCC6MDR cells [7,8]. On the basis of the mechanism of the ABCB1 ‘hydrophobicity vacuum cleaner’ model [11], lipid solubility is the key factor to improve the reverse tumor MDR activity. Therefore, three methoxy groups of the C ring played a significant role in the ABCB1 inhibition effect (Figure 1, shown in red).

2-Aminoimidazole marine alkaloids have various biological activities, including anti-cancer [12], antimicrobial [13,14], antivirus properties [15], ABCB1-mediated MDR reversal activity [16] as well as both leukotriene B4 receptor [17] and epidermal growth factor (EGF) receptor antagonist activities [10]. Lissodendrins B (Figure 1) is a 2-aminoimidazole marine alkaloid first extracted from the EtOAc fraction of the sponge Lissodendoryx (Acanthodoryx) fibrosis in 2016 [18]. The compound was devoid of cytotoxicity, and its total synthesis was performed by our group [19]. OC144-093 reversed multidrug resistance to doxorubicin, paclitaxel, and vinblastine in human lymphoma, breast, ovarian, uterine, and colorectal carcinoma cell lines expressing ABCB1 with an average EC_50_ of 0.032 mM [20]. The structure of Lissodendrins B and the third-generation ABCB1 inhibitors **OC144-093** have similar ‘Y’-type skeleton structural characteristics and may have ABCB1 inhibitory activity to reverse tumor MDR (Figure 1). 

Over the past few decades, three generations of ABCB1 inhibitors have been developed [21,22]. Both the first- and second-generation inhibitors have the drawbacks of poor therapeutic efficacy and high toxicity [23], whereas the third-generation inhibitors (e.g., tariquidar and WK-X-34, Figure 1) show strong ABCB1 inhibitory activities [4,24,25]. Unfortunately, to date, none were practicable in the clinic, mainly because of the limited potency, high inherent toxicity, and adverse drug–drug interactions [26,27,28]. These failures have not concluded the development of novel ABCB1 inhibitors but reveal the evolutionary trends of their structures, where the presence of tetrahydroisoquinoline and benzene rings is important for ABCB1 inhibitors [24,29,30] (Figure 1, shown in blue). Therefore, although the discovery of novel ABCB1 inhibitors with supernal potency and low cytotoxicity is challenging, there is an enormous demand for ABCB1 inhibitors with high efficacy for clinical application.

Based on the structural characteristics of marine products and known third-generation ABCB1 inhibitors, the structure–activity relationships of these compounds reversing ABCB1-meidated drug resistance are as follows: (1) similar ‘Y’-type skeleton structural characteristics may have ABCB1 inhibitory activity; (2) three methoxy groups of C ring, dimethoxy-substituted tetrahydroisoquinoline fragment and benzene rings are the key groups to improve the reversal resistance activity; (3) lipid solubility is the key factor to improve the ABCB1 inhibitory activities. They provide effective guidance for future studies of Lissodendrins B reversing resistance activity through derivatives. In this work, we intended to use 2-aminoimidazole as the ‘Y’ scaffold for the design of a series of Lissodendrins B derivatives. These designed compounds were synthesized, and their ABCB1 inhibitory activities as well as their synergic effects with DOX were evaluated using MCF-7/ADR and K562/ADR cells. Furthermore, we studied the mechanisms underlying the ABCB1 inhibitory activities and revealed that the presentative compounds could enhance the intracellular concentration of DOX via inhibiting the transporting function of ABCB1 rather than its expression.

## 2. Results

A series of Lissodendrins B analogues was designed, in which the 2-positions of 1-methyl-1*H*-imidazole rings were substituted with different functional groups. The key intermediates **7** and **8** were prepared and reported by our groups previously [19,31], as depicted in Scheme S1. Based on the structural characteristics of marine products and the third-generation ABCB1 inhibitors, the numbers of the methoxy group of the C ring, dimethoxy-substituted tetrahydroisoquinoline fragment, benzene rings and the different linkers between the imidazole ring and B ring were the key groups to improve the reversal resistance activity, and the typical moieties were synthesized as follows.

The key intermediates **13** and **14** were synthesized, as illustrated in Scheme S2. For example, compound **11** was prepared via the Williamson reaction from the starting material benzyl bromide in the mixture of CH_3_CN with K_2_CO_3_ for 5 h. Compound **11** was saponified in the presence of LiOH to produce lithium salt, followed by the hydrochloride acidification of compound **13**.

The key intermediates **17** and **18** were synthesized using methods shown depicted in Scheme S3. Compound **15** was prepared via the Mitsunobu reaction from the starting material 3,4-dimethoxymethylbenzoate in the mixture of THF with DIAD and PPh_3_. Compound **15** was saponified in the presence of LiOH to produce lithium salt, followed by the hydrochloride acidification of compound **17**.

The key intermediate **21** was synthesized using the method depicted in Scheme S4. The key intermediate **21** was prepared and reported by our groups previously [31]. Compound **20** was prepared from the starting material 6,7-dimethoxy-1,2,3,4-tetrahydroisoquinoline hydrochloride refluxed in the mixture of CH_3_CN with methyl bromoacetate and potassium carbonate for 8 h. Then, compound **20** with lithium hydroxide in the mixture of THF and H_2_O gave the corresponding lithium carboxylate, which was protonated with hydrochloric acid to generate key intermediate **21**.

The key intermediate **25** was synthesized using the methods described in Scheme S5. Firstly, intermediates **22** and **23** were successfully synthesized, as reported in the literature [29]. Then, compound **24** was prepared from the starting compound **23,** refluxed in the mixture of EtOH with methyl bromoacetate and CH_3_COONa for 24 h. Finally, compound **24** was saponified in the presence of LiOH to produce lithium salt, followed by the hydrochloride acidification of compound **25**.

**Scheme 1:**
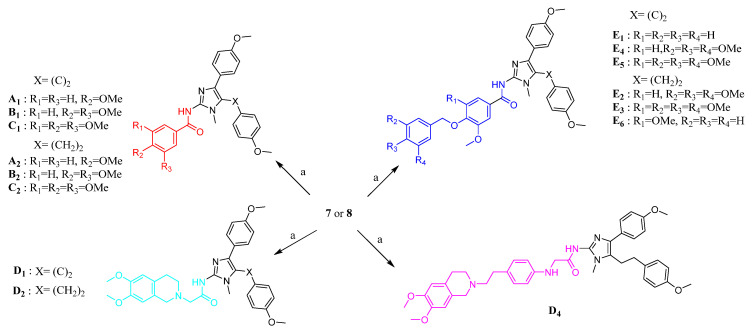
Synthesis of target products. Reagent and conditions: (a) appropriate carboxylic acid, EDCI, HOAt, DIPEA, CH_2_Cl_2_, reflux.

As shown in Scheme 1, the target products could be synthesized through the acylation reaction of compound **7** or **8** with different methoxy-substituted benzoyl acid and tetrahydroisoquinoline derivatives. The compounds **A_1_**–**A_2_**, **B_1_**–**B_2_**, **C_1_**–**C_2_**, **D_1_**–**D_2_**, and **E_1_**–**E_5_** were prepared and reported by our groups previously [31]. Compound **7** or **8** was coupled with compound **14** or **25** to yield, respectively, **D_4_** and **E_6_**, catalyzed by EDCI and HOAt in CH_2_Cl_2_. 

**Scheme 2:**
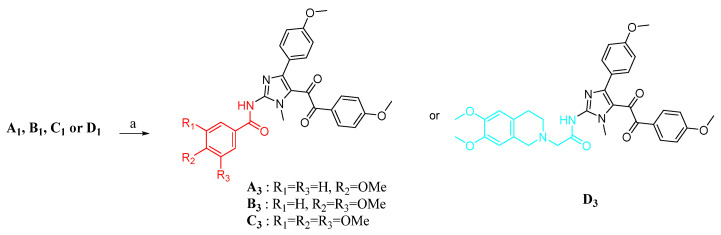
Synthesis of target products. Reagent and conditions: (a) Hg(NO_3_)_2_, DMF, rt.

The synthetic routes for compounds **A_3_**, **B_3_**, **C_3_**, and **D_3_** are shown in Scheme 2. Successive oxidation of **A_1_**, **B_1_**, **C_1_**, and **D_1_** with mercuric nitrate hydrate (2 equiv) gave *α*-diketones compounds **A_3_**, **B_3_**, **C_3_**, and **D_3_**, respectively. The compounds **A_3_**, **B_3_**, **C_3_**, and **D_3_** were prepared and reported by our groups previously [31].

### 2.1. Biological Evaluation 

#### 2.1.1. Biological Investigation and Structure–Activity Relationship Study

For the identification of effective ABCB1 inhibitors, we evaluated the drug resistance reversal effects of these newly synthesized compounds, using DOX-resistant K562/ADR and MCF-7/ADR cells as screening models.

We found that when Lissodendrin B was combined with DOX, the IC_50_ of ABCB1 overexpressed K562/ADR and MCF-7/ADR cells was almost unchanged, and the RF value of Lissodendrin B was about 1, indicating that it did not have an ABCB1 inhibition effect (Table 1). On the basis of the mechanism of the ABCB1 “hydrophobicity vacuum cleaner” model [11], hydrophilic Lissodendrin B was unlikely to enter the hydrophobic drug-binding cavity and, thus, high hydrophobicity was required to improve ABCB1 inhibition activity. A series of Lissodendrin B derivatives with improved hydrophobicity was designed and synthesized, and those compounds showed ABCB1 inhibition activity (Table 1). Therefore, we elaborated on the structure–activity relationship of these compounds afterwards. 

In the ‘Y’-shaped skeleton compounds (**A_1_**–**A_3_**, **B_1_**–**B_3_**, and **C_1_**–**C_3_**), the RF of **A_1_**, **B_1_**, and **C_1_** in K562/ADR cell resistance was 10.60, 21.73, and 26.04, respectively; RF values reversing MCF-7/ADR resistance were 12.61, 17.90, and 7.77, respectively (Table 1). By comparing the RF values of **A_1_**, **B_1_**, and **C_1_**, we found that the ABCB1 inhibition activity was increased with three methoxy groups of the C ring when the linker between the imidazole ring and B ring was unchanged (RF**_C1_** > RF**_B1_** > RF**_A1_**). When the linker was alkyne or ethyl (**A_1_**, **A_2_**) rather than *α*-diketone (**A_3_**) structure, ABCB1 inhibition activity was also improved (RF**_A1_** > RF**_A2_** > RF**_A3_**). Meanwhile, compounds **B_1_**–**B_3_** with the different linkers and three methoxy groups of the C ring also possessed ABCB1 inhibition activities (RF**_B1_** > RF**_B2_** > RF**_B3_**).

When combined with DOX at 10 µM, these compounds (**E_1_**–**E_6_**) with a benzyl group or benzyl substitution could effectively reduce the IC_50_ value of DOX for both cancer cells (Table 1). However, their reversal multiple RF values were less than 15, and the ABCB1 inhibition activities were lower than the compounds (**A_1_**–**A_3_**, **B_1_**–**B_3_**, and **C_1_**–**C_3_**), indicating that the introduction of a benzyl group or benzyl group with three methoxy groups on the C ring was not conducive to improving the ABCB1 inhibition activity of cancer cells.

**Figure 2:**
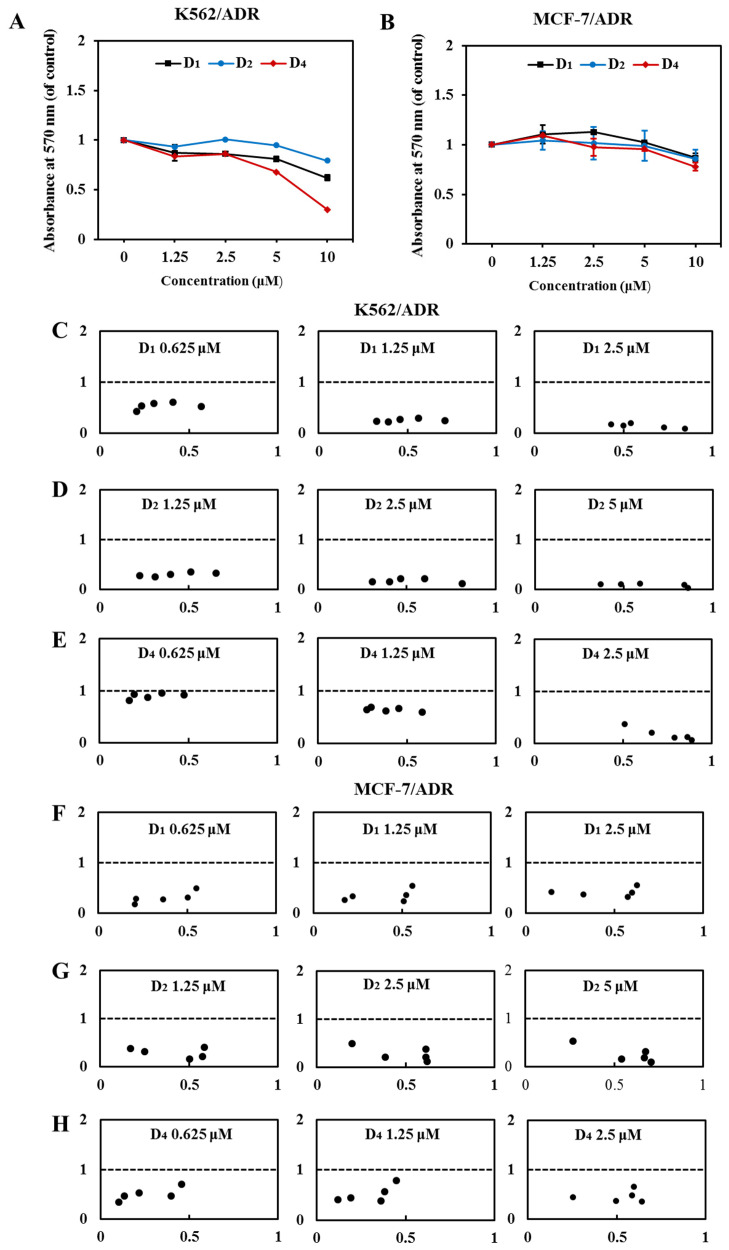
Compounds **D_1_**, **D_2_**, and **D_4_** sensitize the effect of DOX in K562/ADR and MCF-7/ADR cells. (**A**) K562/ADR cells and (**B**) MCF-7/ADR cells were treated with compounds **D_1_**, **D_2_**, and **D_4_** (0–10 μM), respectively, for 72 h. Cell viability was tested via MTT assay, and the value was a viability ratio relative to the control group. Treatment with DOX in the absence and presence of compounds **D_1_**, **D_2_**, and **D_4_** for 72 h in K562/ADR cells (**C**–**E**) and MCF-7/ADR cells (**F**–**H**). The proliferation inhibition was tested via MTT assay, and the CI value was calculated in CompuSyn software. The CI values of >1, =1, and <1 indicate antagonistic, additive, and synergistic effects, respectively.

Compounds **D_1_**–**D_4_** with the pharmacophore-6,7- dimethoxytetrahydroisoquinoline structure were obtained. Our results showed that compounds **D_1_**, **D_2_**, and **D_4_** (10 μM) in combination with DOX for 72 h had higher reversal fold in both K562/ADR and MCF-7/ADR cells (Table 1). At 10 µM, the calculated RF values of K562/ADR and MCF-7/ADR cells were 1845.76 and 207.86 for **D_1_**, 80.00 and 40.63 for **D_2_**, and 1645.45 and 131.67 for **D_4_**, which were higher than other compounds. By comparing the RF values of **A_1_**, **B_1_**, **C_1_**, **D_1_**, and **D_2_**, we found that the ABCB1 inhibition activity of compounds **D_1_** and **D_2_** was observably increased with the pharmacophore-6,7-dimethoxytetrahydroisoquinoline structure compared to the compounds **A_1_**, **B_1_**, and **C_1_** with methoxy groups of the C ring. Meanwhile, compound **D_4_** displayed the most potent ABCB1 inhibition activities with an RF value of 1645.45, which was remarkably higher than compounds **E_1_**–**E_6_** (RF values ≤ 14.3). Compounds **D_1_**, **D_2_**, and **D_4_** with dimethoxy-substituted tetrahydroisoquinoline fragment were the most promising ABCB1 inhibitors, which indicated that the imidazole amino ligation pharmacophore was critical to improve ABCB1 inhibition activities. They provide effective guidance for future studies of Lissodendrins B inhibiting ABCB1 activity through derivatives. It should be noted that the calculated IC_50_ values of DOX and reversal fold in the presence of compounds **D_1_**, **D_2_**, and **D_4_** were partly due to their own cytotoxicity of these compounds, because after the treatment of compounds **D_1_**, **D_2_**, and **D_4_** for 72 h, a higher concentration (5 and 10 μM) of these compounds alone showed moderate cytotoxicity (Figure 2A,B).

#### 2.1.2. Compounds **D_1_**, **D_2_**, and **D_4_** Have a Synergic Effect in Combination with DOX

Next, we investigated the synergic effect of compounds **D_1_**, **D_2_**, and **D_4_** under sub-lethal concentrations. Our results showed that at relatively lower concentrations, 2.5 μM of **D_1_** and **D_4_**, and 5 μM of **D_2_** showed negligible inhibition on the cell viability (Figure 2A,B), and, therefore, we chose these sub-lethal concentrations to further evaluate their synergic combination with DOX and the concentration–response effects. As shown in Table 2, when the concentrations of compounds **D_1_**, **D_2_**, and **D_4_** combined with DOX were decreased, the IC_50_ of DOX on K562/ADR and MCF-7/ADR cells was significantly increased, and the RF values of the compounds were reduced, but they were still greater than one, indicating that they still have MDR reversal activity. Meanwhile, when DOX was combined with different concentrations of **D_1_**, **D_2_**, and **D_4_**, both in K562/ADR and MCF-7/ADR cells, all the CI values were smaller than one and in an obvious concentration-dependent manner (Figure 2C–H), indicating that **D_1_**, **D_2_**, and **D_4_** had strong synergic effects when in combination with DOX. Among these three compounds, compound **D_1_** showed the strongest synergic effect, and **D_1_** was chosen as a representative compound for the following experiments.

#### 2.1.3. Molecular Docking of **D_1_** with ABCB1

**Figure 3:**
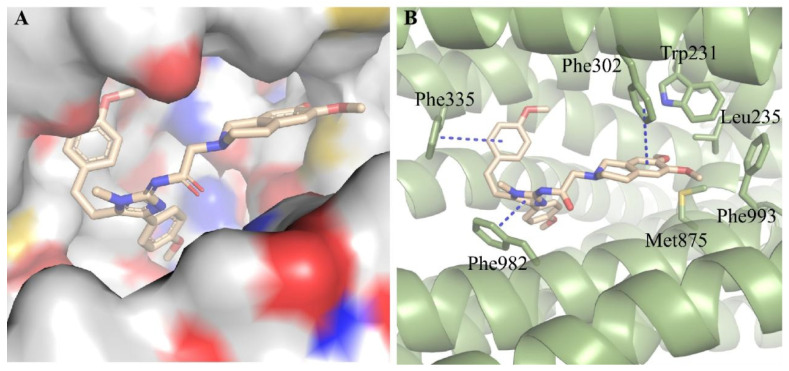
Predicted binding mode of compound **D_1_** with ABCB1 (PDB code: 6FN1). (**A**) Compound **D_1_** well fitted into the hydrophobic cavity of the binding pocket. (**B**) 3D view of the interaction of compound **D_1_** (yellow) with ABCB1 (green, shown as cartoon). The π-π stacking interactions showed in blue dash line.

We subsequently conducted docking analysis to investigate the interacting binding mode of compound **D_1_** with ABCB1 (PDB: 6FN1) [32,33]. The result showed that compound **D_1_** well fitted into the hydrophobic binding pocket formed by Trp231, Leu235, Phe302, Phe335, Phe982, Met875, and Phe993 of ABCB1 (Figure 3A,B). T-π stacking interactions were found between the phenyl ring of compound D1 and Phe335, and between the tetrahydroisoquinoline ring of **D_1_** and Phe302, while π-π stacking was formed between the imidazole ring of **D_1_** and Phe982 (Figure 3B). Meanwhile, the tetrahydroisoquinoline ring of **D_1_** was also stabilized through hydrophobic interaction with the side chains of Trp231, Leu235, Met875, and Phe993. We conducted docking analysis to investigate the interacting binding modes of zosuquidar and compound **D_1_** with ABCB1. We found that zosuquidar and compound **D_1_** had the same binding pocket and similar binding patterns (Figure S1A,B). Meanwhile, the stable binding pattern of compound **D_1_** with ABCB1 was demonstrated by root-mean-square deviation (RMSD) in 50 ns molecular dynamic simulations (Figure S1C). These interactions contribute greatly to the high binding affinity of compound **D_1_** to ABCB1 and provide a fundamental structural basis for the further structure-based design of novel ABCB1 inhibitors.

#### 2.1.4. Compound **D_1_** Increases the Intracellular Accumulation of DOX in K562/ADR and MCF-7/ADR Cells

**Figure 4:**
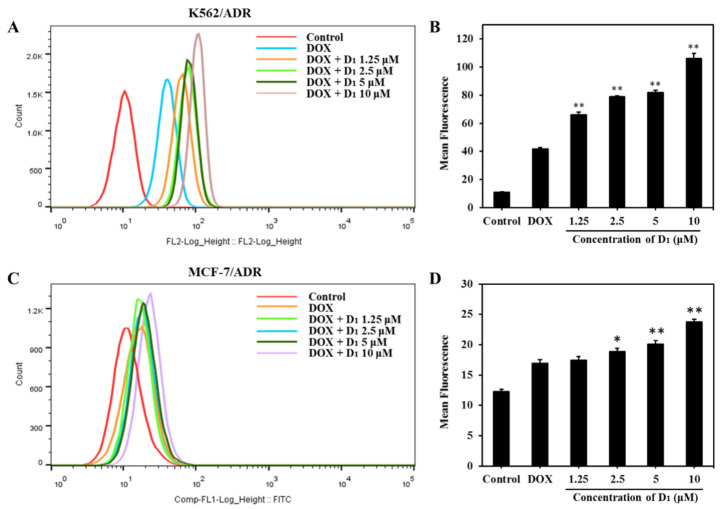
Compound **D_1_** increases the intracellular accumulation of DOX both in K562/ADR and MCF-7/ADR cells. K562/ADR and MCF-7/ADR cells were treated with DOX (3 μM) in the presence or absence of compound **D_1_** (1.25–10 μM) for 12 h. (**A**) Intracellular DOX accumulation in K562/ADR cells was detected through flow cytometry (Beckman Coulter MoFlo XDP). (**B**) The mean of DOX fluorescent intensity in K562/ADR cells shown in the histograms. (**C**) Intracellular accumulation of DOX was detected via flow cytometry, and (**D**) histograms show the mean fluorescence of DOX in MCF-7/ADR cells. Values represent the means ± SD of three experiments. * *p* < 0.05, ** *p* < 0.01.

To further investigate the mechanisms underlying the MDR reversal effect of compound **D_1_**, we analyzed the intracellular accumulation of DOX in K562/ADR and MCF-7/ADR cells using flow cytometry. As shown in Figure 4A,B, a relatively lower concentration of DOX was accumulated in DOX-alone-treated K562/ADR cells. However, in the presence of different concentrations of compound **D_1_**, the accumulation of DOX significantly increased, and the accumulation effect was shown in a concentration-dependent manner. The effect of **D_1_** enhancing intracellular accumulation of DOX was also observed in MCF-7/ADR cells (Figure 4C,D). These results indicate that the mechanisms underlying the reversal effect of **D_1_** are possibly related to the inhibition of the ABCB1 transporting function, as ABCB1 is the major transporter that mediates the DOX efflux and drug resistance.

#### 2.1.5. Compound **D_1_** increases the Intracellular Accumulation of ABCB1 Substrate Rho123 in K562/ADR and MCF-7/ADR Cells

**Figure 5:**
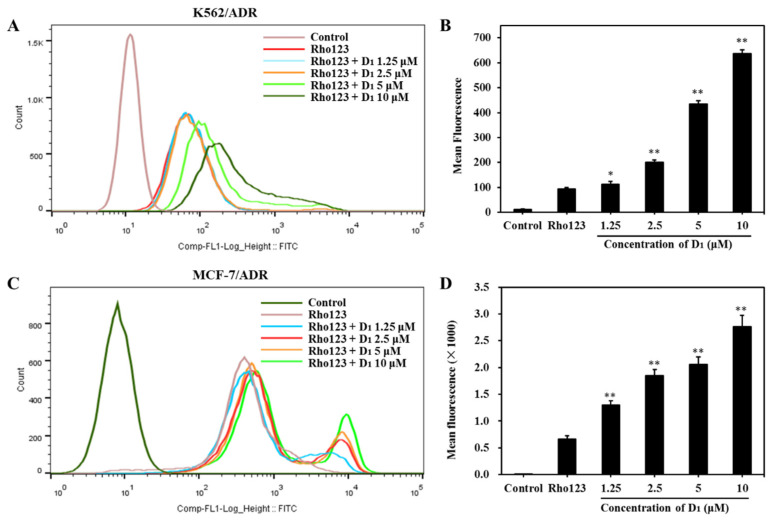
Compound **D_1_** inhibits the efflux of Rho123 both in K562/ADR cells and MCF-7/ADR cells. Cells were treated with compound **D_1_** (1.25–10 μM) for 6 h, then incubation with Rho123 (10 μM) for another 2 h. (**A**) The intracellular accumulation of Rho123 in K562/ADR cells was analyzed via flow cytometry, and (**B**) the mean fluorescence of Rho123 is shown in the histograms. (**C**) The intracellular accumulation of Rho123 in MCF-7/ADR cells was detected via flow cytometry, and (**D**) histograms show the increasing mean fluorescence of Rho123. Values represent the means ± SD of three experiments. * *p* < 0.05, ** *p* < 0.01.

To confirm whether **D_1_** affects the ABCB1 transporting function, we next analyzed the efflux and intracellular accumulation of Rho123, which is a classical substrate of ABCB1. As shown in Figure 5A,B, in the presence of compound **D_1_**, in K562/ADR cells, the intracellular Rho123 fluoresces increased in a concentration-dependent manner. The same phenomenon was also observed in MCF-7/ADR cells (Figure 5C,D), confirming that compound **D_1_** could significantly increase the intracellular accumulation of Rho123. These results further revealed that compound **D_1_** could inhibit the efflux function of ABCB1.

#### 2.1.6. ABCB1 Is Involved in Compound **D_1_**-Mediated MDR Reversal

**Figure 6:**
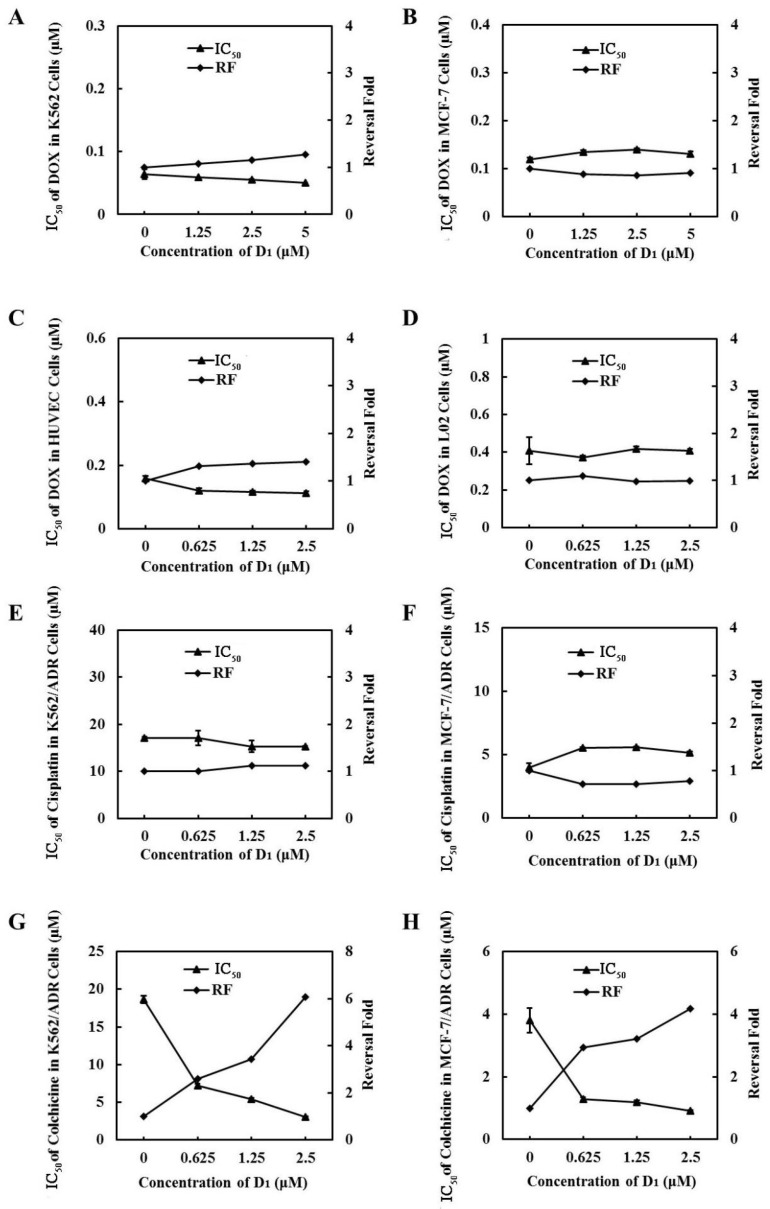
The inhibition effect of MDR is related to ABCB1. Compound **D_1_** does not enhance the sensitization of DOX in MCF-7 cells (**A**), K562 cells (**B**), HUVECs (**C**), and L02 cells (**D**). MCF-7 cells and K562 cells were incubated with DOX in absence or presence of certain concentrations of compound **D_1_** (1.25–5 μM) for 72 h; HUVECs and L02 cells were treated with DOX in absence and presence of compound **D_1_** (0.625–2.5 μM). Cell viability was assayed via MTT method, and the IC_50_ values of DOX and the reversal fold were calculated. Compound **D_1_** does not reverse the resistance of cisplatin in K562/ADR cells (**E**) and MCF-7/ADR cells (**F**). Compound **D_1_** enhances the sensitivity of ABCB1 substrate COL in K562/ADR cells (**G**) and MCF-7/ADR cells (**H**). Cells were treated with cisplatin or COL in absence and presence of compound **D_1_** (0.625–2.5 μM). Cell viability was assayed via MTT method; the IC_50_ values of DOX and the reversal fold were calculated.

To further demonstrate that ABCB1 was involved in compound **D_1_**-mediated MDR reversal, we used DOX-sensitive cell line MCF-7 cells and K562 cells, which express low ABCB1, to evaluate the synergic effect of DOX and **D_1_**. We found that compound **D_1_** did not enhance the sensitivity of DOX in these two DOX-sensitive cell lines (Figure 6A,B). Meanwhile, the synergic effect was not observed in normal human liver L02 cells and human umbilical vein endothelial cells (HUVECs) (Figure 6C,D). Moreover, when combined with cisplatin, which is not a substrate of ABCB1 in K562/ADR and MCF-7/ADR cells, compound **D_1_** did not decrease the IC_50_ of cisplatin and showed no synergic effect (Figure 6E,F). However, when combined with colchicine, which is a substrate of ABCB1, as expected, **D_1_** could significantly enhance the sensitivity of colchicine, both in K562/ADR and in MCF-7/ADR cells (Figure 6G,H). Taken together, compound **D_1_** sensitized the chemotherapeutic agents in MDR cells via inhibition of the efflux function of ABCB1.

#### 2.1.7. Compound **D_1_** Did Not Have an Obvious Effect on the Expression Levels of ABCB1

**Figure 7:**
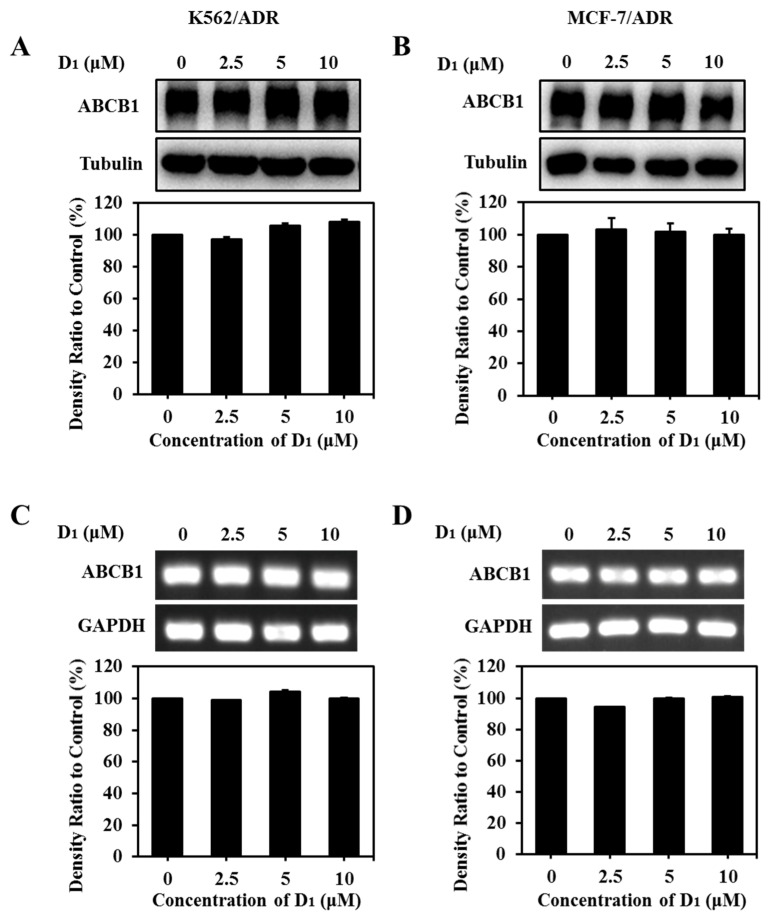
Compound **D_1_** has no significant effect on ABCB1 expression level. Cells were treated with certain concentrations of compound **D_1_** (0–10 μM) for 24 h in K562/ADR cells and MCF-7/ADR cells, respectively. The expression levels of ABCB1 were detected via Western blotting assay, and the histograms show the expression levels of protein bands in K562/ADR cells (**A**) and MCF-7/ADR cells (**B**). The expression levels of ABCB1 mRNA were measured using RT-PCR in K562/ADR cells (**C**) and MCF-7/ADR cells (**D**). Histograms show the relative density ratio to control group.

Both the expression downregulation and the functional inhibition can decrease the ABCB1 transporting function. In order to elucidate the transporting inhibition mechanism of ABCB1, the effect of compound **D_1_** on ABCB1 expression was analyzed via WB assay and RT-PCR assay. The results showed that compound **D_1_** did not obviously alter the protein expression level (Figure 7A,B) or mRNA expression level (Figure 7C,D) of ABCB1 in K562/ADR and MCF-7/ADR cells under the present experimental conditions. The results revealed that reversal MDR of compound **D_1_** was not through the downregulation of ABCB1 expression but rather through inhibiting the transport function of ABCB1.

### 2.2. Discussion

In this study, nineteen Lissodendrins B analogues were synthesized as ABCB1 inhibitors to overcome ABCB1-mediated MDR. A preliminary structure–activity relationship study showed that ‘Y’-type skeleton structural characteristics, three methoxy groups of the C ring, dimethoxy-substituted tetrahydroisoquinoline fragment, and the linker between the imidazole ring and B ring essentially contribute to ABCB1 inhibiting activity. The compounds (**D_1_**, **D_2_**, and **D_4_**) with the pharmacophore-6,7-dimethoxytetrahydroisoquinoline were the most promising ABCB1 inhibitors, which indicated that the tetrahydroisoquinoline as a pharmacophore was critical to improve the ABCB1 inhibition effect. 

Meanwhile, our present work revealed that the presentative compound **D_1_** could significantly reverse the drug resistance of MCF-7/ADR and K562/ADR cells to DOX and indicated that **D_1_** had the potential to develop as a good DOX and other chemotherapeutic agent sensitizer for ABCB1 transporter-mediated MDR in clinical cancer treatment. Parent compounds of **D_1_** usually influence multiple bioactivities, e.g., DNA damage, telomerase inhibition, and senescence induction, and, thus, it is interesting to explore the effect of **D_1_** on other bioactivities since this kind of compound has multiple activities and mechanisms in cancer therapy. A study is ongoing in our laboratory to study its effect on these bioactivities, as well as address the in vivo activity of **D_1_** on xenograft tumor models. 

A high expression of ABCB1 plays a crucial role in chemotherapeutic agent resistance, and targeting ABCB1 has become an promising strategy to reverse MDR [34]. In our previous screening work for ABCB1 inhibitors, we found that cardiotonic steroid compounds display anti-DOX resistance activity by stimulating ABCB1 ATPase activity [35]. Flavonoid compounds, e.g., xanthohumol and isoxanthohumol, also act as ABCB1 substrates and reverse MDR in MCF-7/ADR cells [36,37]. Other research groups also showed that tyrosine kinase inhibitors modulate ABCB1 functions through stimulating the ATPase activity [38]. In this work, we reported that **D_1_** and its derivatives could reverse DOX resistance by inhibiting the efflux function of ABCB1 for the first time. It should be noted that **D_1_**-reversed ABCB1-mediated MDR possibly occurred via the modulation of ABC protein expression. In the following experiments, we then found that up to 24 h, 10 μM **D_1_** did not affect ABCB1 expression, both in mRNA and protein levels, confirming that **D_1_** inhibited the efflux of DOX and was not via modulation of ABCB1 expression level under our present experimental conditions.

Considering that other ABC transporters also contributed to the development of MDR, we could not eliminate the effect of **D_1_** on other ABC transporters, such as ABCG2 and multidrug resistance protein 1 (MRP1). As compound **D_1_** could inhibit the efflux function of ABCB1, it is an interesting topic to further evaluate whether **D_1_** could interfere with the pharmacokinetics of chemotherapeutic agents, e.g., DOX or paclitaxel, which are substrates of ABCB1. Moreover, most of the present ABCB1 inhibitors in clinical trials have failed due to side effects or unpredictable pharmacokinetic drug–drug interactions. Thus, there is still a reason to develop more efficacious and non-toxic ABCB1 inhibitors in order to reverse MDR in cancer treatment [39]. 

## 3. Materials and Methods

### 3.1. Chemical Design and Synthesis Materials

Dichloromethane and tetrahydrofuran were purified conventionally. All reagents used in the experiments were obtained from commercial sources without further purification. Reactions were monitored through thin-layer chromatography (TLC). Visualization was achieved under a UV lamp (254 nm and 365 nm) and developed the plates with potassium permanganate. Flash column chromatography was performed with silica gel (200–300 mesh). ^1^H NMR and ^13^C NMR spectra were taken on JnmEcp-600 spectrometer. ^1^H NMR and ^13^C NMR spectra are cell lines referenced to tetramethylsilane (Me4Si). The following abbreviations are used: s = singlet, d = doublet, t = triplet, q = quartet, m = multiplet, dd = double-doublet.

### 3.2. Biological Evaluation

#### 3.2.1. Cell Culture and Reagents

Cells: Human breast cancer MCF-7 cells and the adriamycin-resistant cell line MCF-7/ADR, human myelogenous leukemia K562 cell lines and adriamycin-resistant cell line K562/ADR, human hepatacyte L02 cells, and Human umbilical vein endothelial cells (HUVECs) were obtained from the Cell Bank of Chinese Academy of Sciences. K562 cells, K562/ADR cells, and L02 cells were maintained in Iscove’s Modified Dulbecco’s Medium (IMDM) supplemented with 10% fetal bovine serum (FBS). MCF-7 cells were maintained in Roswell Park Memorial Institute (RPMI)-1640 medium supplemented with 10% FBS. MCF-7/ADR cells were maintained in Eagles Minimum Essential Medium (EMEM) supplemented with 10% FBS. HUVECs were maintained in Dulbecco’s Modified Eagle Medium (DMEM) supplemented with 10% FBS. All media were supplemented with antibiotics (100 IU/mL penicillin G, and 100 μg/mL streptomycin). All cell lines were maintained at 37 °C in humidified 5% CO_2_ incubator. Reagents: Antibody against ABCB1 was from Cell Signaling Technology (Boston, MA, USA). Antibodies against β-tubulin were purchased from Epitomics (Burlingame, CA, USA). The RNA extraction kit was purchase from Tiangen (Beijing, China). The PrimeScriptTM RT-PCR kit was purchased from Takara (Kusatsu, Shiga, Japan). The other reagents were purchased from Beyotime Biotechnology (Shanghai, China).

#### 3.2.2. Detection of Cell Proliferation

The effect of compounds on cell proliferation was determined by 3-(4,5-dimethylthiazol-2-yl)-2,5-diphenyltetrazolium bromide (MTT) assay. Cells were seeded in 96-plate wells and incubated with the indicated concentrations of compounds for indicated periods. Then, MTT (20 μL, 5 mg/mL) was added in each well. After incubation at 37 °C for another 4 h, supernatant of adherent cells was discarded; then, 150 μL DMSO was added and incubated for about 15 min. Meanwhile, acidic isopropanol 100 μL was added into the wells of suspension cells, and plates were further incubated overnight to dissolve the formazan product. Finally, absorbance was measured at 570 nm.

The sulforhodamine B (SRB) assay was another method used to detect the effect of compounds on cell growth. Briefly, cells were seeded in 96-well plates and treated for indicated time with various concentrations of compounds. Then, the supernatant liquid was discarded; cells were immobilized by ice-cold trichloroacetic acid (TCA, 10%) for 1 h and washed with deionized water. After drying at 37 °C, 100 μL 0.4% SRB was added in every well and incubated for 15 min. SRB was discarded and washed with 1% glacial acetic acid. After drying at 37 °C, 150 μL tris-base (10 mM) was added in every well and incubated for 10 min, and the absorbance was measured at 492 nm.

#### 3.2.3. Calculation of Combined Drug Synergistic Effects

Cells were seeded in 96-well plates and treated with DOX alone or combined with certain concentrations of tested compounds for 72 h; then, the cell viability was detected using MTT method as mentioned above. The drug combination index (CI) was calculated in CompuSyn software (ComboSyn, Inc., Paramus, NJ, USA) according to the description of Chou and Talalay [40]. The CI values of >1, =1, and <1 indicate antagonistic, additive, and synergistic effects, respectively.

#### 3.2.4. Intracellular DOX Accumulation Assay

Cells were seeded in 6-well plates and treated with DOX (3 μM) alone or in presence of compound **D_1_** (0–10 μM) for 12 h, and then cells were collected and washed three times with ice-cold phosphate buffered saline (PBS). The autofluoresence of DOX was detected via flow cytometer (Beckman Coulter MoFlo XDP, Fullerton, CA, USA) with an excitation wavelength of 488 nm.

#### 3.2.5. Intracellular Rhodamine 123 (Rho123) Accumulation Assay

Cells were pre-treated with different concentrations of compound **D_1_** (0–10 μM) for 6 h. Rho123 (10 μM) was added in the culture medium for another 2 h. Then, the cells were collected and washed 3 times with chilled PBS. Cells were resuspended in 500 μL PBS and analyzed via flow cytometry (Beckman Coulter MoFlo XDP, Fullerton, CA, USA). The excitation wavelengths of Rho 123 were 488 nm, and the emission wavelength was 530 nm.

#### 3.2.6. ABCB1 mRNA Level and RT-PCR

Cells were seeded in 6-well plates and treated with certain concentrations of compound **D_1_** (0–10 μM) for 24 h. After incubation, total RNA was obtained via RNA extraction kit according to the manufacturer’s instruction. Then, the RT-PCR was performed with PrimeScriptTM RT-PCR kit. ABCB1 mRNA was measured using the following primers [36]: ABCB1 FP: 5′-CCCATCATTGCAATAGCAGG-3′; RP: 5′-GTTCAAACTTCTGCTCCTGA-3′. The primer pairs for GAPDH were: FP: 5′-GAAGGTGAAGGTCGGAGTC-3′; RP: 5′-GAAGATGGTGATGGGATTTC-3′. The electrophoresis of the products running on 1% agarose gels contained gel red. The results were detected via Gel DocTM XR + (BIO-RAD, Heracles, CA, USA) and analyzed with Image LabTM Software (BIO-RAD, Heracles, CA, USA).

#### 3.2.7. Western Blotting Assay

Cells were seeded in 6-well plates and treated with certain concentrations of compound D1 (0–10 μM) for 24 h. Then, cells were collected, washed twice with PBS, and lysed with loading buffer for 45 min at 4 °C. The total protein samples were separated by 6–8% SDS polyacrylamide gel electrophoresis (PAGE) and immobilized on the nitrocellulose membrane through electroblotting. The membranes were blocked in 5% milk for 2 h and incubated with specific primary antibodies at 4 °C overnight, followed by incubation with secondary antibodies for 1 h. Finally, protein bands were visualized using enhanced chemiluminescence kits and quantified by Tanon-5200 (Tanon, Shanghai, China).

#### 3.2.8. Statistical Analysis

The results were given as mean ± SD, and Student’s *t*-test was employed for the statistical comparisons. *p* < 0.05 was considered as significant difference compared with the control group.

## 4. Conclusions

In summary, a series of 19 Lissodendrins B analogues was synthesized, and the ABCB1-mediated MDR reversal activity in DOX-resistant K562/ADR and MCF-7/ADR cells was evaluated. Amongst them, we revealed that these novel compounds **D_1_**, **D_2_**, and **D_4_** possessed potent synergistic effects with DOX and reversed ABCB1-mediated drug resistance, which demonstrated that the introduction of a dimethoxy-substituted tetrahydroisoquinoline fragment was a successful strategy for the development of ABCB1 inhibitors. Notably, compound **D_1_** exhibited low cytotoxicity and the strongest synergistic effect. The synergistic mechanisms were mainly related to the increased intracellular accumulation of DOX, via inhibiting the efflux function of ABCB1 rather than from affecting the expression level of ABCB1. Considering the potent MDR reversal activity and the relatively safe profiles, compound **D_1_**, as a potentially promising lead, deserves further investigation for developing ABCB1 inhibitors to overcome ABCB1-mediated MDR.

## Figures and Tables

**Table 1:** IC_50_ values of DOX and the calculated theoretically reversal fold (RF) in the absence and presence of compounds (10 μM) on K562/ADR and MCF-7/ADR cells.

Compounds (10 μM)	IC_50_ (DOX, µM)	RF	IC_50_ (DOX, µM)	RF
	K562/ADR		MCF-7/ADR	
DOX	14.48 ± 1.37		19.75 ± 1.96	
**A_1_**	1.37 ± 0.01	10.60	1.57 ± 0.01	12.61
**A_2_**	2.69 ± 0.21	5.39	2.56 ± 0.05	7.71
**A_3_**	2.08 ± 0.08	6.97	5.70 ± 0.00	3.46
**B_1_**	0.67 ± 0.05	21.73	1.10 ± 0.04	17.90
**B_2_**	0.96 ± 0.15	15.10	1.73 ± 0.03	11.44
**B_3_**	3.83 ± 0.02	3.78	4.77 ± 0.02	4.14
**C_1_**	0.56 ± 0.01	26.04	2.54 ± 0.21	7.77
**C_2_**	0.21 ± 0.00	68.95	1.05 ± 0.04	18.86
**C_3_**	1.86 ± 0.39	7.77	6.81 ± 0.15	2.90
**D_1_**	0.0078 ± 0.00	1845.76	0.10 ± 0.03	207.86
**D_2_**	0.18 ± 0.01	80.00	0.49 ± 0.06	40.63
**D_3_**	0.91 ± 0.03	15.89	4.05 ± 0.00	4.87
**D_4_**	0.0088 ± 0.00	1645.45	0.15 ± 0.00	131.67
**E_1_**	1.52 ± 0.04	9.56	4.92 ± 0.02	4.02
**E_2_**	1.01 ± 0.00	14.30	3.32 ± 0.04	5.95
**E_3_**	1.65 ± 0.03	8.80	3.56 ± 0.02	5.55
**E_4_**	6.70 ± 0.00	2.16	6.75 ± 0.10	2.93
**E_5_**	1.92 ± 0.03	7.54	3.71 ± 0.03	5.33
**E_6_**	1.69 ± 0.02	8.59	3.54 ± 0.05	5.58
Lissodendrin B	17.23 ± 0.80	0.84	15.57 ± 0.01	1.27

**Table 2:** IC_50_ values of DOX and the reversal fold (RF) in the absence and presence of compounds **D_1_**, **D_2_**, and **D_4_** on K562/ADR and MCF-7/ADR cells.

Compounds	Concentration (μM)	IC_50_ (DOX)	RF	IC_50_ (DOX)	RF
		K562/ADR		MCF-7/ADR	
DOX		14.54 ± 0.97		20.20 ± 1.31	
**D_1_**	2.50	0.35 ± 0.01	41.54	3.54 ± 0.12	15.71
	1.25	2.70 ± 0.01	5.39	5.12 ± 0.14	3.95
	0.63	8.13 ± 0.05	1.79	6.49 ± 0.07	3.11
**D_2_**	5.00	1.23 ± 0.01	11.87	1.73 ± 0.20	11.66
	2.50	2.18 ± 0.00	6.67	2.91 ± 0.05	6.94
	1.25	4.28 ± 0.01	3.40	4.21 ± 0.04	4.80
**D_4_**	2.50	0.48 ± 0.00	30.45	2.61 ± 0.28	7.73
	1.25	5.91 ± 0.23	2.46	10.59 ± 0.15	1.91
	0.63	13.52 ± 0.50	1.08	11.05 ± 0.18	1.83

## Data Availability

The authors declare that the supporting data of this study are available within the article and the Supplementary Materials.

## Supplementary Materials

The following supporting information can be downloaded at: https://www.mdpi.com/article/10.3390/md21050314/s1, General procedure for synthesis of compounds **11**–**18**, **20** and **21**. General Procedure for the Synthesis of Compounds **A_1_**, **A_2_**, **B_1_**, **B_2_**, **C_1_**, **C_2_**, **D_1_**, **D_2_**, **E**_1_–**E**_5_. Scheme S1: Chemical synthesis of compounds **7** and **8**. Scheme S2: Chemical synthesis of compounds **13** and **14**. Scheme S3: Chemical synthesis of compounds **17** and **18**. Scheme S4: Chemical synthesis of compound **21**. Scheme S5: Chemical synthesis of compound **25**. Figure S1: Predicted binding modes of zosuquidar and compound **D_1_** with ABCB1. Figures S2–S39: Copies of 1H NMR and 13C NMR Spectra of Compounds **A_1_**–**A_3_**, **B_1_**–**B_3_**, **C_1_**–**C_3_**, **D_1_**–**D_4_** and **E_1_**–**E_6_**.
