# Supplementary material for: Design, Synthesis, and Biological Evaluation of Marine Lissodendrins B Analogues as Modulators of ABCB1-Mediated Multidrug Resistance
Source: Mar Drugs. 2023 May 20;21(5):314. doi: 10.3390/md21050314 (PMC10220702; doi:10.3390/md21050314)
Supplement: Supplementary file 1 [file marinedrugs-21-00314-s001.zip › marinedrugs-2377884-supplementary-highlight.pdf]

Supporting information

Design, synthesis, and biological evaluation of marine Lissodendrins B analogues as modulators of ABCB1-mediated multidrug resistance

Chaoming Wang [1, 2,†], Jinman Zhang [1,†], Xianfeng Wei [1,†], Mengke Yang[1, 2], Weiping Ma [1], Rilei Yu [1, 2, *], Ming Liu [1, 2,*] , and Tao Jiang [1, 2,]

[1]Key Laboratory of Marine Drugs, The Ministry of Education of China, School of Medicine and Pharmacy, Ocean University of China, Qingdao 266003, China ; wangchaoming315@163.com (C.W.); hdszhjm@126.com (J.Z.); weixfeng427@163.com (X.W.); yangmengke@stu.ouc.edu.cn （M.Y.）; maweiping1990@163.com (W.M.)

[2]Laboratory for Marine Drugs and Bioproducts and Innovation Center for Marine Drug Screening & Evaluation, Qingdao National Laboratory for Marine Science and Technology, Qingdao, 266003, China

*Correspondence: ryu@ouc.edu.cn (R.Y.); lmouc@ouc.edu.cn (M.L.); jiangtao@ouc.edu.cn (T.J.)

† These authors contributed equally to this work.

Contents of this PDF

1. General procedure for synthesis of compounds **11-18**, **20** and **21** 3

2. General Procedure for the Synthesis of Compounds **A$_1$**, **A$_2$**, **B$_1$**, **B$_2$**, **C$_1$**, **C$_2$**, **D$_1$**, **D$_2$**, **E$_1$-E$_5$**. 5

3. Scheme S1. Chemical synthesis of compounds **7** and **8** 10

4. Scheme S2. Chemical synthesis of compounds **13** and **14** 10

5. Scheme S3. Chemical synthesis of compounds 1**7** and 1**8** 11

6. Scheme S4. Chemical synthesis of compound **21** 11

7. Scheme S5. Chemical synthesis of compound **25** 11

8. Figure S1. Predicted binding modes of zosuquidar and compound **D$_1$** with ABCB1

.... 12

9. Copies of ^{1}H NMR and ^{13}C NMR Spectra of Compounds **A_1-A_3**, **B_1-B_3**, **C_1-C_3**, **D_1-D_4** and **E_1-E_6** 13

1. General procedure for synthesis of compounds **11-18**, **20** and **21**

To a stirring solution of compound **9** (5 g, 27.4 mmol) and K_2CO_3 (18.9 g, 137 mmol) in CH_3CN (60 mL) was added benzyl bromide (7.0 g, 41.1 mmol). After stirring at reflux for 5 h, the solvent was removed under a vacuum. The residue was poured into water and extracted with ethyl acetate. The organic layers were washed with water, brine, dried, and concentrated. The residue was purified by silica gel column chromatography to give compounds **11** and **12**.

methyl 4-(benzyloxy)-3-methoxybenzoate (**11**). White solid, Yield: 59.0%, ^1H NMR (400 MHz, $CDCl_3$) δ 7.61 (dd, J = 8.4, 2.0 Hz, 1H), 7.56 (d, J = 2.0 Hz, 1H), 7.45 – 7.30 (m, 5H), 6.89 (d, J = 8.4 Hz, 1H), 5.21 (s, 2H), 3.93 (s, 3H), 3.88 (s, 3H); ^{13}C NMR (100 MHz, $CDCl_3$) δ 166.8, 152.1, 149.1, 136.4, 128.6, 128.1, 127.2, 123.3, 122.9, 112.4, 112.4, 70.7, 56.1, 52.1.

methyl 4-(benzyloxy)-3,5-dimethoxybenzoate (**12**). White solid, Yield: 60.6%, ^1H NMR (400 MHz, $CDCl_3$) δ 7.49 – 7.44 (m, 2H), 7.36 – 7.27 (m, 5H), 5.09 (s, 2H), 3.90 (s, 3H), 3.87 (s, 6H); ^{13}C NMR (100 MHz, $CDCl_3$) δ 166.7, 153.2, 140.9, 137.3, 128.4, 128.2, 128.0, 125.3, 106.7, 74.9, 56.2, 52.2.

To a stirring solution of compound **10** (5.4 g, 25.2 mmol), DIAD (10.2 g, 50.4 mmol) and PPh_3 (13.3 g, 50.4 mmol) in THF (50 mL) was added 3,4,5-trimethoxybenzyl alcohol (5 g, 25.2 mmol). After stirring for 5 h at room temperature, the solvent was removed under a vacuum. The residue was poured into water and extracted with ethyl acetate. The organic layers were washed with water, brine, dried, and concentrated. The residue was purified by silica gel column chromatography to give compounds **15** and **16**.

methyl 3-methoxy-4-((3,4,5-trimethoxybenzyl)oxy)benzoate (**15**). White solid, Yield: 46.1%, ^1H NMR (400 MHz, $CDCl_3$) δ 7.64 (dd, J = 8.4, 1.9 Hz, 1H), 7.59 (s, 1H), 6.94 (d, J = 8.4 Hz, 1H), 6.68 (s, 2H), 5.14 (s, 2H), 3.96 (s, 3H), 3.91 (s, 3H), 3.87 (s, 6H), 3.85 (s, 3H); ^{13}C NMR (100 MHz, $CDCl_3$) δ 166.8, 153.5, 152.1, 149.1, 137.8, 132.0, 123.4, 123.1, 112.6, 112.4, 104.4, 71.2, 60.8, 56.1, 56.1, 52.0.

Methyl 3,5-dimethoxy-4-((3,4,5-trimethoxybenzyl)oxy)benzoate (**16**). White solid, Yield: 40.4%, ^1H NMR (400 MHz, $CDCl_3$) δ 7.32 (s, 2H), 6.75 (s, 2H), 5.05 (s, 2H),

3.93 (s, 3H), 3.91 (s, 6H), 3.88 (s, 6H), 3.85 (s, 3H); ^{13}C NMR (100 MHz, $CDCl_3$) δ 166.7, 153.1, 153.1, 140.9, 137.7, 133.1, 125.3, 106.7, 105.3, 75.2, 60.8, 56.2, 56.0, 52.2.

Compound **13**, **14**, **17**, **18** were synthesized by the same method. To a stirring solution of the corresponding compounds (**11**, **12**, **15** or **16**, 1 mmol) in THF (20 mL) and water (20 mL) was added LiOH (10 mmol) at 0 ◦C. After stirring for 8 h at room temperature, the solvent was removed under vacuum. The residue was poured into water and was acidified with 1 N HCl to pH 2-3. The aqueous solution was concentrated to dryness under reduced pressure to give the crude product. (Without purification to the next step).

methyl 2-(6,7-dimethoxy-3,4-dihydroisoquinolin-2 (1H)-yl)acetate (**20**). To a stirring solution of compound **19** (5 g, 21.8 mmol) and K_2CO_3 (7.5 g, 54.5 mmol) in CH_3CN (100 mL) was added methyl bromoacetate (4 g, 26.1 mmol). After stirring at reflux for 8 h, the solvent was removed under a vacuum. The residue was poured into water and extracted with ethyl acetate. The organic layers were washed with water, brine, dried, and concentrated. The residue was purified by silica gel column chromatography to give compound **20** (4.5 g, 77.6%). ^{1}H NMR (400 MHz, $CDCl_3$) δ 6.59 (s, 1H), 6.50 (s, 1H), 3.83 (dd, J = 6.6, 1.7 Hz, 6H), 3.75 (s, 3H), 3.71 (s, 2H), 3.42 (s, 2H), 2.86 (s, 4H); ^{13}C NMR (100 MHz, $CDCl_3$) δ 170.9, 147.5, 147.2, 125.9, 125.6, 111.3, 109.3, 58.9, 55.8, 55.8, 54.9, 51.7, 50.7, 28.3.

2-(6,7-dimethoxy-3,4-dihydroisoquinolin-2(1H)-yl)acetic acid (**21**). To a stirring solution of the corresponding compound **20** (1 mmol) in THF (20 mL) and water (20 mL) was added LiOH (10 mmol) at 0 ◦C. After stirring for 8 h at room temperature, the solvent was removed under vacuum. The residue was poured into water and was acidified with 1 N HCl to pH 2-3. The aqueous solution was concentrated to dryness under reduced pressure to give the crude product **21**. (Without purification to the next step).

2. General Procedure for the Synthesis of Compounds $\mathbf{A_1}$, $\mathbf{A_2}$, $\mathbf{B_1}$, $\mathbf{B_2}$, $\mathbf{C_1}$, $\mathbf{C_2}$, $\mathbf{D_1}$, $\mathbf{D_2}$, $\mathbf{E_1}$-$\mathbf{E_5}$.

To a stirring solution of the corresponding carboxylic acid (1.2 mmol) in dichloromethane (20 mL) was added EDCI (1.2 mmol), HOAt (1.2 mmol), and DIPEA (3 mL) at room temperature. After stirring for 2 h at room temperature, compound (7 or 8, 0.6 mmol) was added. After stirring at 45°C for 6 h, saturated aqueous $NaHCO_3$ was added and extracted with CH_2Cl_2. The organic layers were washed with water, brine, dried, and concentrated. The residue was purified by silica gel column chromatography to give the corresponding compounds ($\mathbf{A_1}$, $\mathbf{A_2}$, $\mathbf{B_1}$, $\mathbf{B_2}$, $\mathbf{C_1}$, $\mathbf{C_2}$, $\mathbf{D_1}$, $\mathbf{D_2}$, $\mathbf{E_1}$-$\mathbf{E_5}$).

4-methoxy-N-(4-(4-methoxyphenyl)-5-((4-methoxyphenyl)ethynyl)-1-methyl-1H-imidazol-2-yl)benzamide ($\mathbf{A_1}$). Yield: 42.8%, white solid, ^{1}H NMR (400 MHz, $CDCl_3$) δ 8.21 (d, J = 8.2 Hz, 2H), 7.86 (d, J = 8.1 Hz, 2H), 7.49 (d, J = 8.2 Hz, 2H), 7.01-6.88 (m, 6H), 3.86 (d, J = 5.0 Hz, 9H), 3.69 (s, 3H). ^{13}C NMR (100 MHz, $CDCl_3$) δ 162.2, 160.3, 159.7, 133.0, 130.5, 126.7, 114.3, 114.3, 114.2, 113.3, 98.7, 55.4, 55.4, 55.3, 29.7; HRMS calcd for $(C_{28}H_{26}O_4N_3+ H)^+$468.1918, found 468.1911.

3,4-dimethoxy-N-(4-(4-methoxyphenyl)-5-((4-methoxyphenyl)ethynyl)-1-methyl-1H-imidazol-2-yl)benzamide ($\mathbf{B_1}$). White solid, Yield: 38.1%, ^{1}H NMR (400 MHz, $CDCl_3$) δ 7.85 (m, 4H), 7.49 (m, 2H), 6.96 (m, 2H), 6.91 (m, 3H), 3.96 (s, 3H), 3.94 (s, 3H), 3.85 (s, 6H), 3.70 (s, 3H). ^{13}C NMR (101 MHz, $CDCl_3$) δ 160.3, 159.8, 151.7, 148.5, 133.1, 126.7, 122.2, 114.4, 114.3, 114.1, 111.3, 110.2, 98.8, 55.9, 55.9, 55.4, 29.7; HRMS calcd for $(C_{29}H_{28}O_5N_3+H)^+$ 498.2023, found 498.2012.

3,4,5-trimethoxy-N-(4-(4-methoxyphenyl)-5-((4-methoxyphenyl)ethynyl)-1-methyl-1H-imidazol-2-yl)benzamide ($\mathbf{C_1}$). white solid, Yield: 38.4%, ^{1}H NMR (400 MHz, $CDCl_3$) δ 7.83 (d, J = 8.9 Hz, 2H), 7.56 (s, 2H), 7.49 (d, J = 8.8 Hz, 2H), 6.96 (d, J = 8.9 Hz, 3H), 6.92 (d, J = 8.8 Hz, 3H), 3.93 (s, 6H), 3.89 (s, 3H), 3.84 (d, J = 1.7 Hz, 6H), 3.70 (s, 3H). ^{13}C NMR (100 MHz, $CDCl_3$) δ 160.3, 159.8, 152.8, 140.8, 133.1,

126.7, 114.4, 114.3, 114.0, 105.8, 98.9, 60.9, 56.1, 55.4, 55.4, 29.7; HRMS calcd for $(C_{30}H_{30}O_6N_3 + H)^+$ 528.2129, found 528.2115.

4-methoxy-N-(5-(4-methoxyphenethyl)-4-(4-methoxyphenyl)-1-methyl-1H-imidazol-2-yl)benzamide (**A$_2$**). white solid, Yield: 34.6%, ^{1}H NMR (400 MHz, $CDCl_3$) δ 8.27 (d, J = 8.8 Hz, 2H), 7.23 (d, J = 8.8 Hz, 2H), 7.03 (d, J = 8.6 Hz, 2H), 6.94 (t, J = 8.1 Hz, 4H), 6.82 (d, J = 8.5 Hz, 2H), 3.88 (s, 3H), 3.86 (s, 3H), 3.80 (s, 3H), 3.55 (s, 3H), 2.98 (t, J =7.6 Hz, 2H), 2.88 (t, J = 7.4 Hz, 2H). ^{13}C NMR (100 MHz, $CDCl_3$) δ 161.8, 159.4, 158.4, 131.8, 131.1, 130.5, 129.4, 128.1, 121.7, 121.1, 114.4, 114.1, 113.1, 55.3, 55.3, 55.3, 34.5, 28.8, 25.8; HRMS calcd for $(C_{28}H_{30}O_4N_3 + H)^+$ 472.2231, found 472.2218.

3,4-dimethoxy-N-(5-(4-methoxyphenethyl)-4-(4-methoxyphenyl)-1-methyl-1H-imidazol-2-yl)benzamide (**B$_2$**). white solid, Yield:31.2%, ^{1}H NMR (400 MHz, $CDCl_3$) δ 7.94 (dd, J = 8.3, 1.9 Hz, 1H), 7.83 (d, J = 1.9 Hz, 1H), 7.20 (d, J = 8.8 Hz, 2H), 6.99 (d, J = 8.6 Hz, 2H), 6.89 (dd, J =8.7, 1.4 Hz, 3H), 6.79 (d, J = 8.6 Hz, 2H), 3.96 (s, 3H), 3.92 (s, 3H), 3.82 (s, 3H), 3.76 (s, 3H), 3.52 (s, 3H), 2.95 (m, 2H), 2.85 (d, J = 7.3 Hz, 2H). ^{13}C NMR (100 MHz, $CDCl_3$) δ 174.2, 159.4, 158.4, 151.2, 148.3, 131.8, 131.3, 129.4, 128.0, 122.1, 121.7, 121.2, 114.4, 114.1, 111.4, 110.1, 55.9, 55.8, 55.3, 55.3, 34.5, 28.8, 25.8; HRMS calcd for $(C_{29}H_{32}O_5N_3 + H)^+$ 502.2336, found 502.2322.

3,4,5-trimethoxy-N-(5-(4-methoxyphenethyl)-4-(4-methoxyphenyl)-1-methyl-1H-imidazol-2-yl)benzamide (**C$_2$**). white solid, Yield: 42%, ^{1}H NMR (400 MHz, $CDCl_3$) δ 7.59 (s, 2H), 7.21 (d, J = 8.8 Hz, 2H), 7.00 (d, J = 8.6 Hz, 2H), 6.91 (d, J = 8.7 Hz, 2H), 6.80 (d, J = 8.6 Hz, 2H), 3.95 (s, 6H), 3.90 (s, 3H), 3.84 (s, 3H), 3.78 (s, 3H), 3.53 (s, 3H), 2.97 (t, J =7.7 Hz, 2H), 2.86 (t, J = 7.4 Hz, 2H). ^{13}C NMR (100 MHz, $CDCl_3$) δ 174.1, 159.4, 158.4, 152.6, 150.3, 140.4, 133.9, 131.7, 129.4, 128.0, 121.5, 121.3, 114.4, 114.1, 105.8, 60.9, 59.5, 56.1, 55.4, 55.3, 34.5, 29.7, 28.8, 25.7; HRMS calcd for $(C_{30}H_{34}O_6N_3 + H)^+$ 532.2442, found 532.2428.

2-*(6,7-dimethoxy-3,4-dihydroisoquinolin-2(1H)-yl)-N-(4-(4-methoxyphenyl)-5-((4-methoxyphenyl)ethynyl)-1-methyl-1H-imidazol-2-yl)acetamide* (**D$_1$**). white solid, Yield: 35%, ^{1}H NMR (400 MHz, $CDCl_3$) δ 8.02 (d, J = 8.9 Hz, 2H), 7.48 (d, J = 8.8 Hz, 2H), 6.92 (dd, J = 8.8, 5.9 Hz, 4H), 6.63 (s, 1H), 6.54 (s, 1H), 3.86 (s, 3H), 3.85 (s,

3H), 3.85 (s, 3H), 3.82 (s, 3H), 3.79 (s, 2H), 3.63 (s, 3H), 3.38 (s, 2H), 2.94 (dd, *J* =11.5, 4.8 Hz, 4H). ^{13}C NMR (100 MHz, $CDCl_3$) *δ* 159.9, 159.0, 151.7, 147.9, 147.5, 139.8, 135.2, 132.9, 129.5, 127.1, 125.3, 121.0, 114.9, 114.2, 113.8, 111.4, 109.3, 103.8, 98.1, 61.2, 56.0, 55.9, 55.8, 55.4, 55.2, 51.7, 31.8, 28.6; HRMS calcd for $(C_{33}H_{35}O_5N_4 + H)^+$ 567.2602, found 567.2586.

3-(6,7-dimethoxy-3,4-dihydroisoquinolin-2(1H)-yl)-N-(5-(4-methoxyphenethyl)-4-(4-methoxyphenyl)-1-methyl-1H-imidazol-2-yl) acetamide (**D_2**). white solid, Yield: 53%, ^1H NMR (400 MHz, $CDCl_3$) *δ* 7.40 (d, *J* = 8.8 Hz, 2H), 7.03 (d, *J* = 8.6 Hz, 2H), 6.88 (d, *J* = 8.8 Hz, 2H), 6.80 (d, *J* = 8.6 Hz, 2H), 6.60 (s, 1H), 6.51 (s, 1H), 3.84 (s, 3H), 3.83 (s, 3H), 3.81 (s, 3H), 3.77 (s, 3H), 3.76 (s, 2H), 3.37 (s, 2H), 3.34 (s, 3H), 2.95 (m, 4H), 2.95 (m, 4H). ^{13}C NMR (100 MHz, $CDCl_3$) *δ* 158.5, 158.2, 147.7, 147.4, 132.5, 129.3, 128.2, 125.6, 125.5, 114.0, 113.9, 111.4, 109.3, 55.9, 55.9, 55.7, 55.3, 55.3, 51.7, 34.7, 30.4, 28.7, 26.6; HRMS calcd for $(C_{33}H_{39}O_5N_4 + H)^+$571.2915, found 571.2898.

4-(benzyloxy)-3-methoxy-N-(4-(4-methoxyphenyl)-5-((4-methoxyphenyl)ethynyl)-1-methyl-1H-imidazol-2-yl)benzamide (**E_1**). white solid, Yield: 29%, ^1H NMR (400 MHz, $CDCl_3$) *δ* 7.84 (m, 4H), 7.49 (d, *J* = 8.8 Hz, 2H), 7.44 (m, 2H), 7.37 (m, 2H), 7.31 (m, 1H), 6.97 (d, *J* = 8.8 Hz, 3H), 6.92 (m, 3H), 5.22 (s, 2H), 3.96 (s, 3H), 3.85 (d, *J* = 1.6 Hz, 6H), 3.68 (s, 3H); ^{13}C NMR (100 MHz, $CDCl_3$) *δ* 160.3, 159.7, 150.9, 149.0, 136.8, 133.0, 128.6, 127.9, 127.3, 126.7, 122.0, 114.3, 114.3, 112.6, 111.8, 98.8, 70.8, 56.0, 55.4, 55.4, 50.8, 29.7; HRMS calcd for $(C_{35}H_{32}O_5N_3 + H)^+$ 574.2336, found 574.2323.

3-methoxy-N-(5-(4-methoxyphenethyl)-4-(4-methoxyphenyl)-1-methyl-1H-imidazol-2-yl)-4-((3,4,5-trimethoxybenzyl)oxy)benzamide (**E_2**). white solid, Yield: 20%, ^1H NMR (400 MHz, $CDCl_3$) *δ* 7.91 (m, 2H), 7.23 (d, *J* = 8.8 Hz, 2H), 7.03 (d, *J* = 8.6 Hz, 2H), 6.98 – 6.89 (m, 3H), 6.82 (d, *J* = 8.6 Hz, 2H), 6.71 (s, 2H), 5.17 (s, 2H), 4.01 (s, 3H), 3.88 (s, 6H), 3.86 (s, 6H), 3.80 (s, 3H), 3.55 (s, 3H), 2.98 (t, *J* = 7.1 Hz, 2H), 2.88 (t, *J* = 7.4 Hz, 2H); ^{13}C NMR (100 MHz, $CDCl_3$) *δ* 159.4, 158.4, 153.4, 150.3, 148.9, 137.6, 132.7, 132.1, 131.8, 129.4, 128.0, 122.0, 121.6, 121.1, 114.4, 114.1,

112.9, 111.9, 104.3, 71.2, 60.8, 56.1, 55.9, 55.3, 55.3, 34.5, 28.8, 25.7; HRMS calcd for $(C_{38}H_{42}O_8N_3 + H)^+$ 668.2966, found 668.2951.

3,5-dimethoxy-N-(5-(4-methoxyphenethyl)-4-(4-methoxyphenyl)-1-methyl-1H-imidazol-2-yl)-4-((3,4,5-trimethoxybenzyl)oxy)benzamide (**E$_3$**). white solid, Yield: 20.2%, ^{1}H NMR (400 MHz, $CDCl_3$) δ 7.62 (s, 2H), 7.24 (d, J = 8.7 Hz, 2H), 7.03 (d, J = 8.6 Hz, 2H), 6.94 (d, J = 8.7 Hz, 2H), 6.82 (d, J = 8.6 Hz, 2H), 6.79 (s, 2H), 5.05 (s, 2H), 3.96 (s, 6H), 3.89 (s, 6H), 3.86 (s, 3H), 3.85 (s, 3H), 3.80 (s, 3H), 3.56 (s, 3H), 2.99 (t, J = 7.4 Hz, 2H), 2.88 (t, J = 7.3 Hz, 2H); ^{13}C NMR (100 MHz, $CDCl_3$) δ 174.1, 159.5, 158.4, 153.0, 152.8, 139.2, 137.5, 134.1, 133.6, 131.7, 129.4, 128.0, 121.4, 121.3, 114.4, 114.1, 105.8, 105.4, 75.2, 60.8, 56.1, 56.0, 55.4, 55.3, 34.5, 28.8, 25.7; HRMS calcd for $(C_{39}H_{44}O_9N_3 + H)^+$ 698.3072, found 698.3052.

3-methoxy-N-(4-(4-methoxyphenyl)-5-((4-methoxyphenyl)ethynyl)-1-methyl-1H-imidazol-2-yl)-4-((3,4,5-trimethoxybenzyl)oxy)benzamide (**E$_4$**). white solid, Yield: 22.6%, ^{1}H NMR (400 MHz, $CDCl_3$) δ 7.85 (d, J = 8.6 Hz, 4H), 7.50 (d, J = 8.7 Hz, 2H), 6.97 (d, J = 8.8 Hz, 2H), 6.93 (m, 3H), 6.68 (s, 2H), 5.14 (s, 2H), 3.97 (s, 3H), 3.86 (d, J = 2.6 Hz, 12H), 3.84 (s, 3H), 3.69 (d, J = 1.5 Hz, 3H); ^{13}C NMR (100 MHz, $CDCl_3$) δ 160.3, 159.8, 153.4, 150.8, 149.1, 137.6, 133.1, 132.5, 126.7, 122.1, 114.4, 114.3, 114.1, 112.8, 111.8, 104.3, 98.8, 77.2, 71.2, 60.8, 56.1, 56.0, 55.4, 55.4; HRMS calcd for $(C_{38}H_{38}O_8N_3 + H)^+$ 664.2653, found 664.2645.

3,5-dimethoxy-N-(4-(4-methoxyphenyl)-5-((4-methoxyphenyl)ethynyl)-1-methyl-1H-imidazol-2-yl)-4-((3,4,5-trimethoxybenzyl)oxy)benzamide (**E$_5$**). white solid, Yield: 20.7%, ^{1}H NMR (400 MHz, $CDCl_3$) δ 7.84 (d, J = 8.8 Hz, 2H), 7.58 (s, 2H), 7.50 (d, J = 8.8 Hz, 2H), 6.98 (d, J = 8.8 Hz, 2H), 6.93 (d, J = 8.8 Hz, 2H), 6.77 (s, 2H), 5.03 (s, 2H), 3.93 (s, 6H), 3.87 (s, 6H), 3.86 (s, 6H), 3.83 (s, 3H), 3.72 (s, 3H); ^{13}C NMR (100 MHz, $CDCl_3$) δ 160.4, 159.9, 153.0, 153.0, 137.6, 133.5, 133.1, 126.6, 114.4, 114.3, 114.0, 105.9, 105.3, 99.0, 77.2, 75.3, 60.8, 56.1, 56.0, 55.4, 55.5; HRMS calcd for $(C_{39}H_{40}O_9N_3 + H)^+$ 694.2759, found 694.2755.

*4.1.4. General Procedure for the Synthesis of Compounds **A$_3$**, **B$_3$**, **C$_3$** and **D$_3$**.*

To a stirring solution of the corresponding compound (**A$_1$**, **B$_1$**, **C$_1$** or **D$_1$**, 0.2 mmol) in DMF (20 mL) was added $Hg(NO_3)_2$.1/2 H_2O (0.4 mmol) at room temperature.

After stirring for 2 h, the solvent was removed under vacuum. The residue was poured into water and extracted with ethyl acetate. The organic layers were washed with water, brine, dried, and concentrated. The crude residue was purified by silica gel column chromatography to give the corresponding compound (**A**$_3$, **B**$_3$, **C**$_3$ or **D**$_3$).

4-methoxy-N-(4-(4-methoxyphenyl)-5-(2-(4-methoxyphenyl)-2-oxoacetyl)-1-methyl-1H-imidazol-2-yl)benzamide (**A**$_3$). Yellow solid, Yield: 55%, ^{1}H NMR (400 MHz, $CDCl_3$) δ 7.94 (d, J = 8.5 Hz, 2H), 7.70 (d, J = 8.7 Hz, 2H), 7.11 (d, J = 8.3 Hz, 2H), 6.90 (m, 2H), 6.80 (d, J =8.5 Hz, 2H), 6.61 (d, J = 8.4 Hz, 2H), 3.93 (s, 3H), 3.91 (s, 3H), 3.84 (s, 3H), 3.75 (s, 3H). ^{13}C NMR (100 MHz, $CDCl_3$) δ 190.0, 185.3, 164.5, 162.8, 160.7, 132.0, 131.2, 130.5, 126.2, 113.9, 113.6, 113.5, 58.4, 55.6, 55.4, 55.3, 18.4; HRMS calcd for $(C_{28}H_{26}O_6N_3 + H)^+$ 500.1816, found 500.1806.

3,4-dimethoxy-N-(4-(4-methoxyphenyl)-5-(2-(4-methoxyphenyl)-2-oxoacetyl)-1-methyl-1H-imidazol-2-yl)benzamide (**B**$_3$). Yellow solid, Yield: 42%, ^{1}H NMR (400 MHz, $CDCl_3$) δ 7.69 (d, J = 8.9 Hz, 4H), 7.09 (d, J = 8.7 Hz, 2H), 6.88 (d, J = 8.9 Hz, 2H), 6.81 (d, J = 8.4 Hz, 1H), 6.60 (d, J = 8.6 Hz, 2H), 3.97 (s, 3H), 3.92 (s, 3H), 3.92 (s, 3H), 3.89 (s, 3H), 3.73 (s, 3H). ^{13}C NMR (100 MHz, $CDCl_3$) δ 189.8, 185.3, 164.5, 160.9, 152.4, 148.7, 132.0, 131.2, 126.2, 122.2, 113.9, 113.6, 111.3, 110.2, 56.0, 55.9, 55.6, 55.3; HRMS calcd for $(C_{29}H_{28}O_7N_3 + H)^+$ 530.1922, found 530.1921.

3,4,5-trimethoxy-N-(4-(4-methoxyphenyl)-5-(2-(4-methoxyphenyl)-2-oxoacetyl)-1-methyl-1H-imidazol-2-yl)benzamide (**C**$_3$). white solid, Yield: 40%, ^{1}H NMR (400 MHz, $CDCl_3$) δ 7.71 (d, J = 8.9 Hz, 2H), 7.51 (s, 2H), 7.12 (d, J = 8.7 Hz, 2H), 6.90 (d, J = 8.9 Hz, 2H), 6.64 (d, J =8.6 Hz, 2H), 4.03 (s, 3H), 3.94 (s, 3H), 3.93 (s, 3H), 3.92 (s, 3H), 3.91 (s, 3H), 3.76 (s, 3H). ^{13}C NMR (100 MHz, $CDCl_3$) δ 189.6, 185.3, 164.6, 161.1, 152.9, 132.0, 131.3, 131.1, 126.1, 114.0, 113.7, 105.9, 60.9, 56.2, 55.6, 55.3, 29.7; HRMS calcd for $(C_{30}H_{30}O_8N_3 + H)^+$ 560.2027, found 560.2012.

3-*(6,7-dimethoxy-3,4-dihydroisoquinolin-2(1H)-yl)-N-(4-(4-methoxyphenyl)-5-(2-(4-methoxyphenyl)-2-oxoacetyl)-1-methyl-1Himidazol-2-yl)acetamide* (**D**$_3$). yellow solid, Yield: 38%, ^{1}H NMR (400 MHz, $CDCl_3$) δ 7.69 (d, J = 8.7 Hz, 2H), 7.09 (d, J = 8.5 Hz, 2H), 6.87 (d, J = 8.9 Hz, 2H), 6.64 (s, 1H), 6.57 (d, J = 8.5 Hz, 2H), 6.54 (s, 1H), 3.93 (s, 3H), 3.89 (s, 3H), 3.88 (s, 3H), 3.86 (s, 3H), 3.81 (s, 2H), 3.72 (s, 3H),

3.44 (s, 2H), 2.97 (m, 4H). ^{13}C NMR (100 MHz, $CDCl_3$) δ 190.1, 185.6, 164.4, 147.9, 147.5, 132.0, 131.1, 126.4, 125.1, 113.8, 113.2, 111.4, 109.3, 61.1, 55.9, 55.9, 55.7, 55.6, 55.2, 51.7, 34.5, 29.7; HRMS calcd for $(C_{33}H_{35}O_7N_4 + H)^+$ 599.2500, found 599.2483.

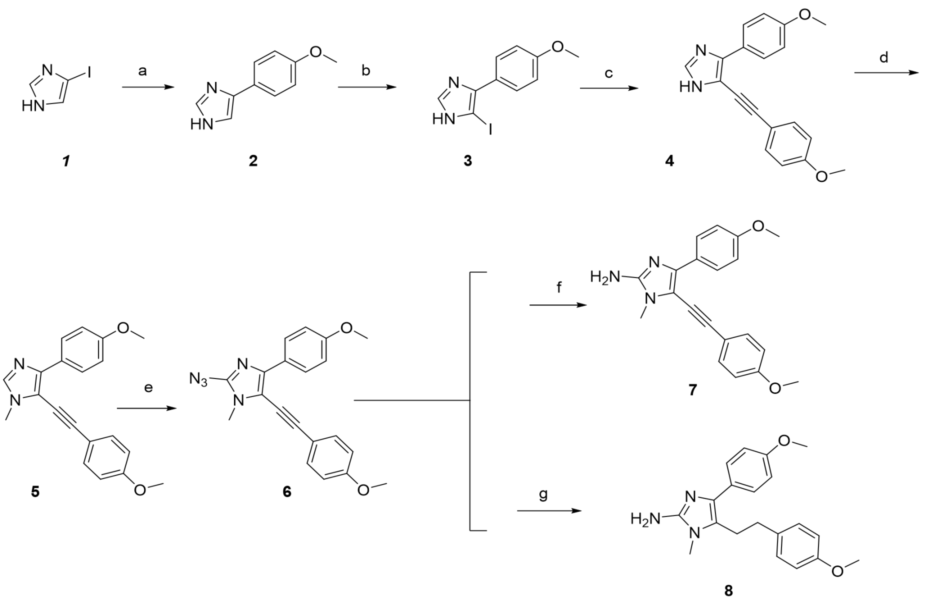

Scheme S1. Chemical synthesis of compounds **7** and **8**. Reagent and conditions: (a) 4-methoxyphenylboronic acid, $Pd(PPh_3)_4$, CsF, Toluene, H_2O, 100°C; (b) NIS, CH_2Cl_2, rt; (c) 4-methoxyphenylacetylene, $Pd(PPh_3)_4$, CuI, TEA, DMF, 80°C; (d) CH_3I, K_2CO_3, DMF, rt; (e) n-C_4H_9Li, TsN_3, THF, -78°C; (f) $Na_2S{\cdot}9H_2O$, CH_3OH, rt; (g) Pd/C, H_2, CH_3OH.

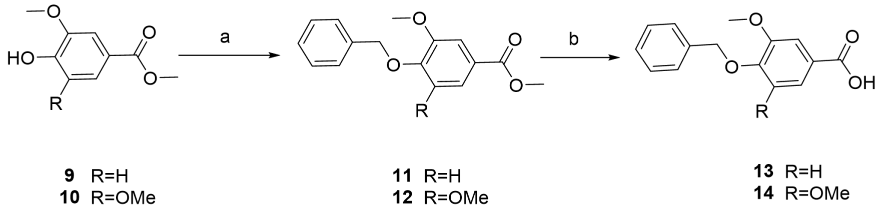

Scheme S2. Chemical synthesis of compounds **13** and **14**. (a) benzyl bromide, K_2CO_3, CH_3CN, rt; (b) 1) LiOH, CH_3OH/H_2O, rt, 2) HCl, H_2O.

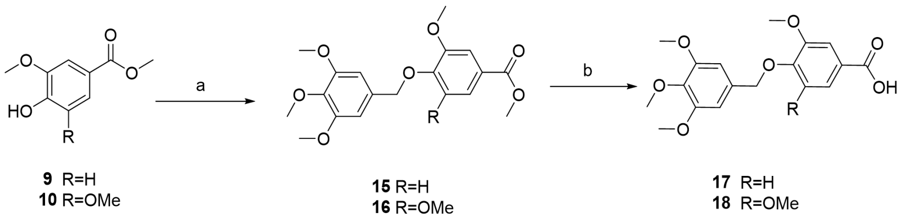

Scheme S3. Chemical synthesis of compounds **17** and **18**. (a) 3,4,5-trimethoxybenzyl alcohol, DIAD, PPh_3, THF, rt; (b) 1) LiOH, CH_3OH/H_2O, rt, 2) HCl, H_2O.

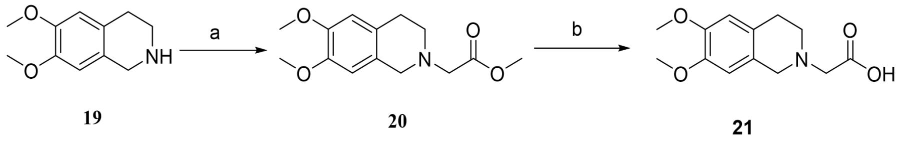

Scheme S4. Chemical synthesis of compound **21**. Reagent and conditions: (a) methyl bromoacetate, K_2CO_3, CH_3CN, reflux; (b) 1) LiOH, THF/H_2O, 2) HCl, H_2O.

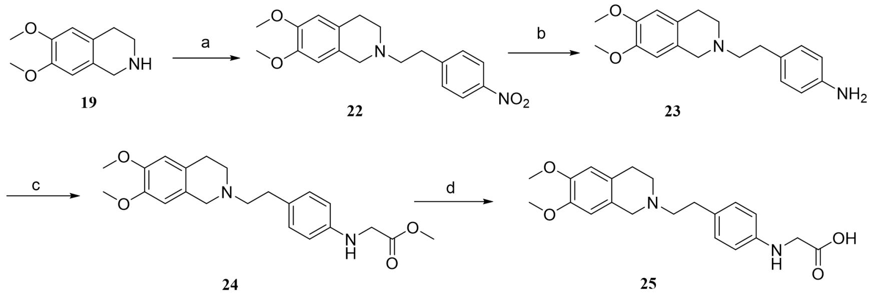

Scheme S5. Chemical synthesis of compound **25**. (a) 4-nitrophenethyl Bromide, K_2CO_3, CH_3CN, reflux, 4 h; (b) Pd/C, H_2, THF/CH_3OH, 8 h, rt; (c) methyl bromoacetate, CH_3COONa, EtOH, reflux, 8 h; (d) 1) LiOH, THF/H_2O, 4 h, 2) HCl, H_2O.

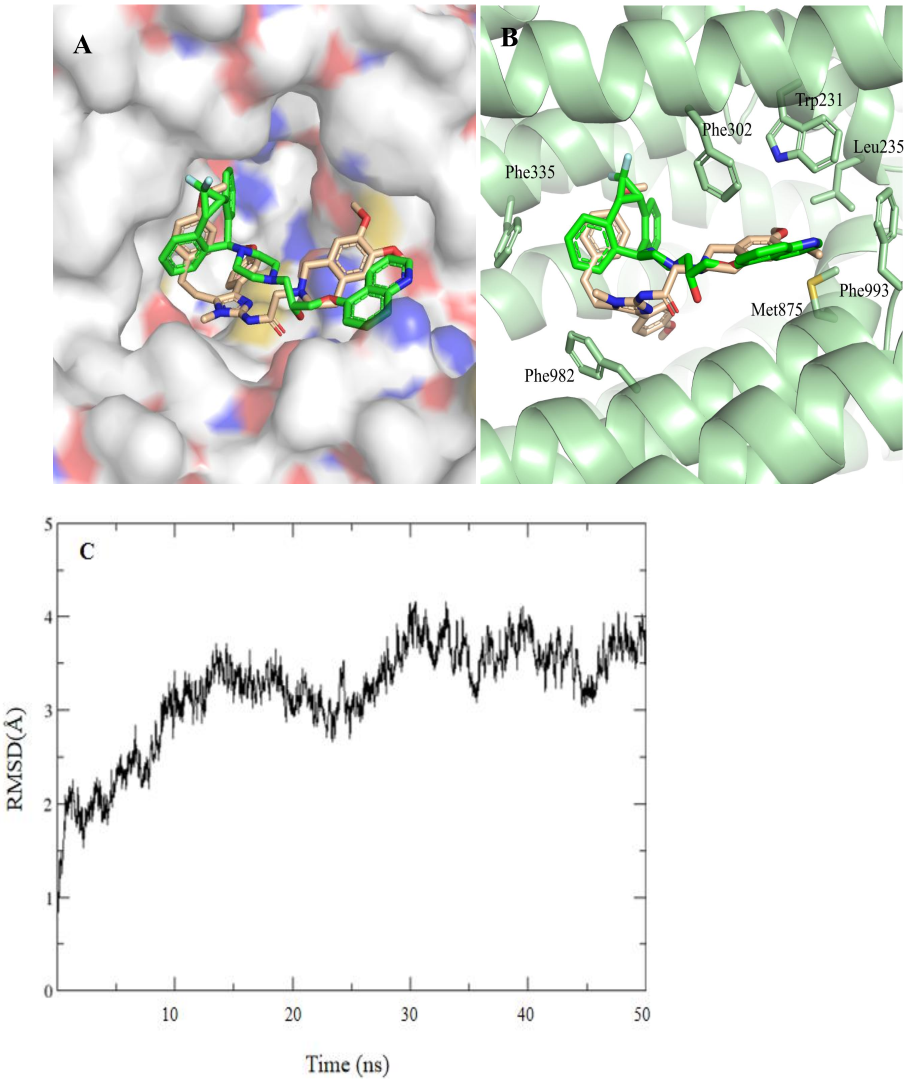

Figure S1. Predicted binding modes of zosuquidar and compound **D_1** with ABCB1 (PDB code: 6FN1 and 7A6F). (A) Zosuquidar and compound **D_1** well fitted into the hydrophobic cavity of the binding pocket. (B) 3D view of the interaction of zosuquidar (green) and compound **D_1** (yellow) with ABCB1. (C) The RMSD (50 ns) of compound **D_1** with ABCB1.

Copies of ^{1}H NMR and ^{13}C NMR Spectra of Compounds A_1-A_3, B_1-B_3, C_1-C_3, D_1-D_4 and E_1-E_6

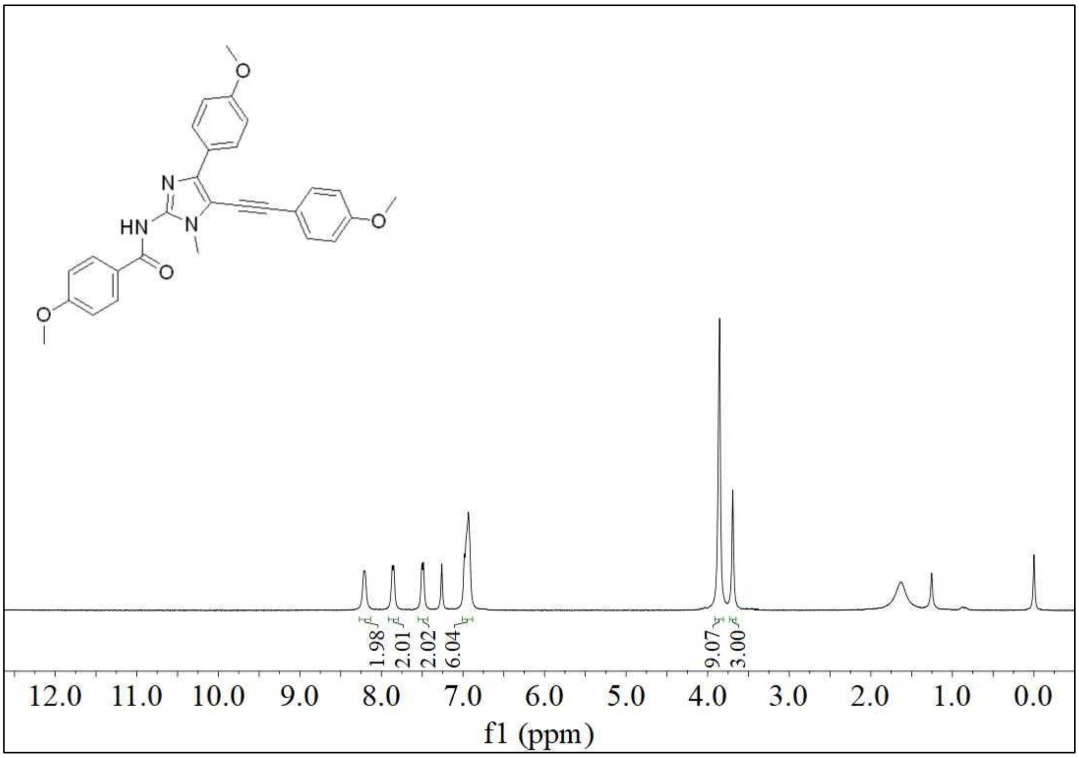

Figure S2. ^{1}H NMR Spectrum of $\mathbf{A_1}$ in $CDCl_3$

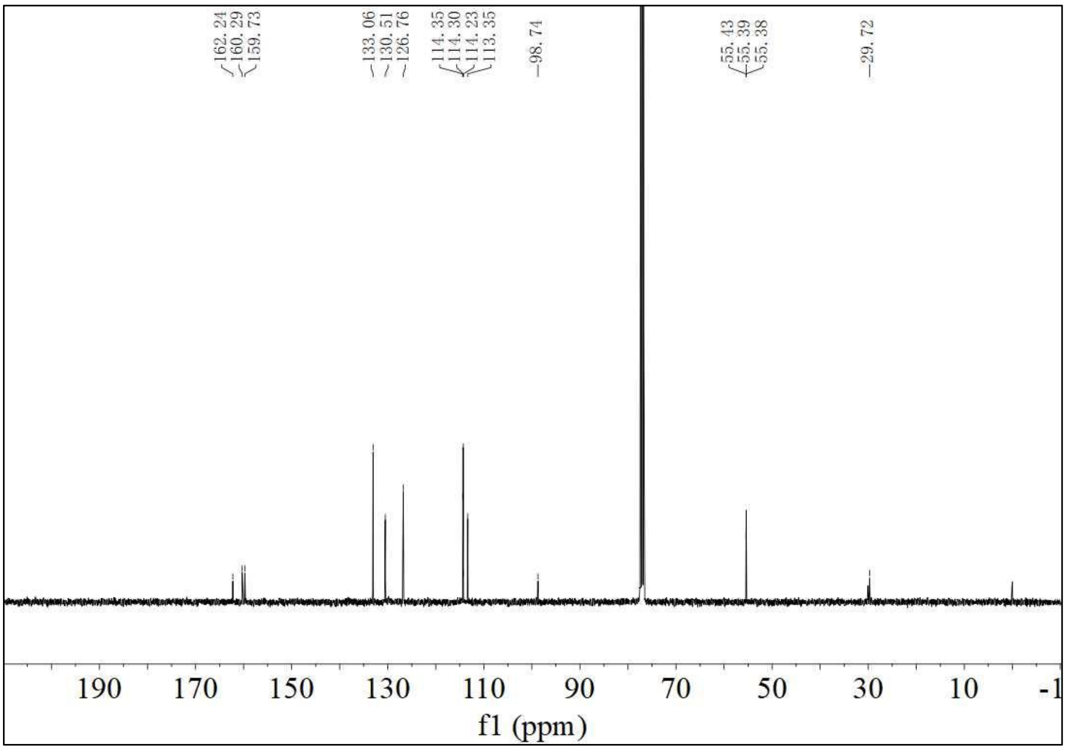

Figure S3. ^{13}C NMR Spectrum of $\mathbf{A_1}$ in $CDCl_3$

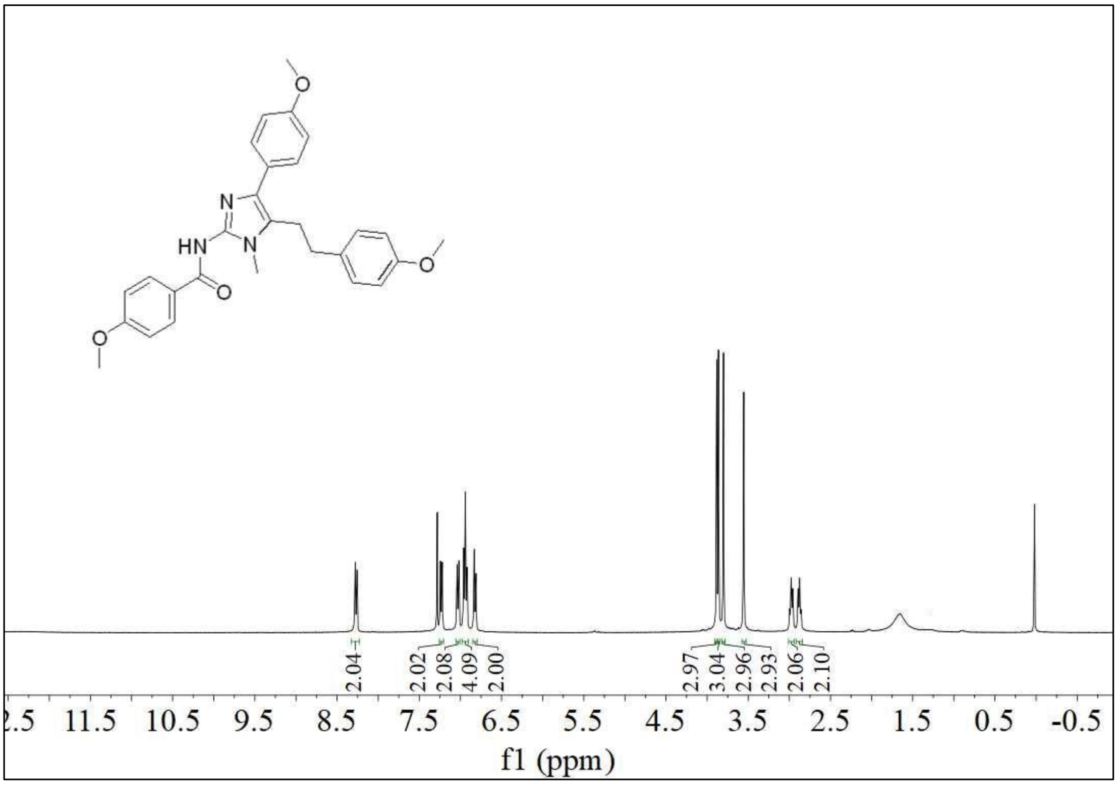

Figure S4. ^{1}H NMR Spectrum of **A$_2$** in $CDCl_3$

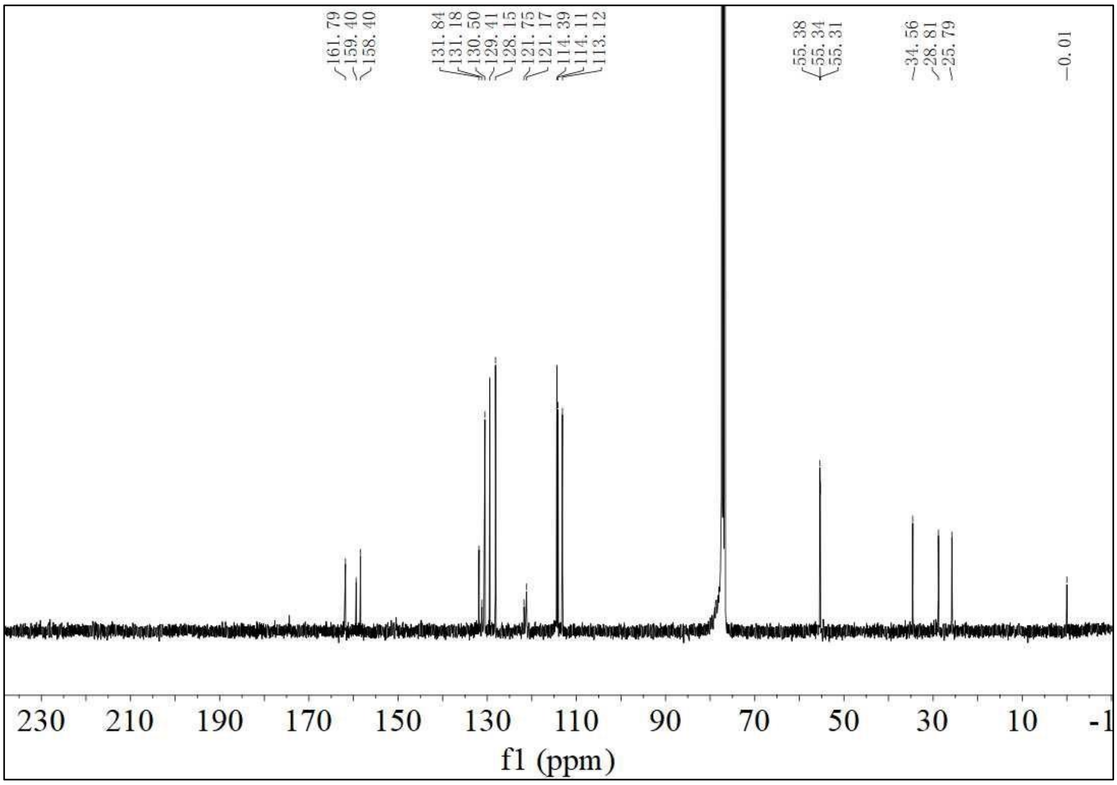

Figure S5. ^{13}C NMR Spectrum of **A$_2$** in $CDCl_3$

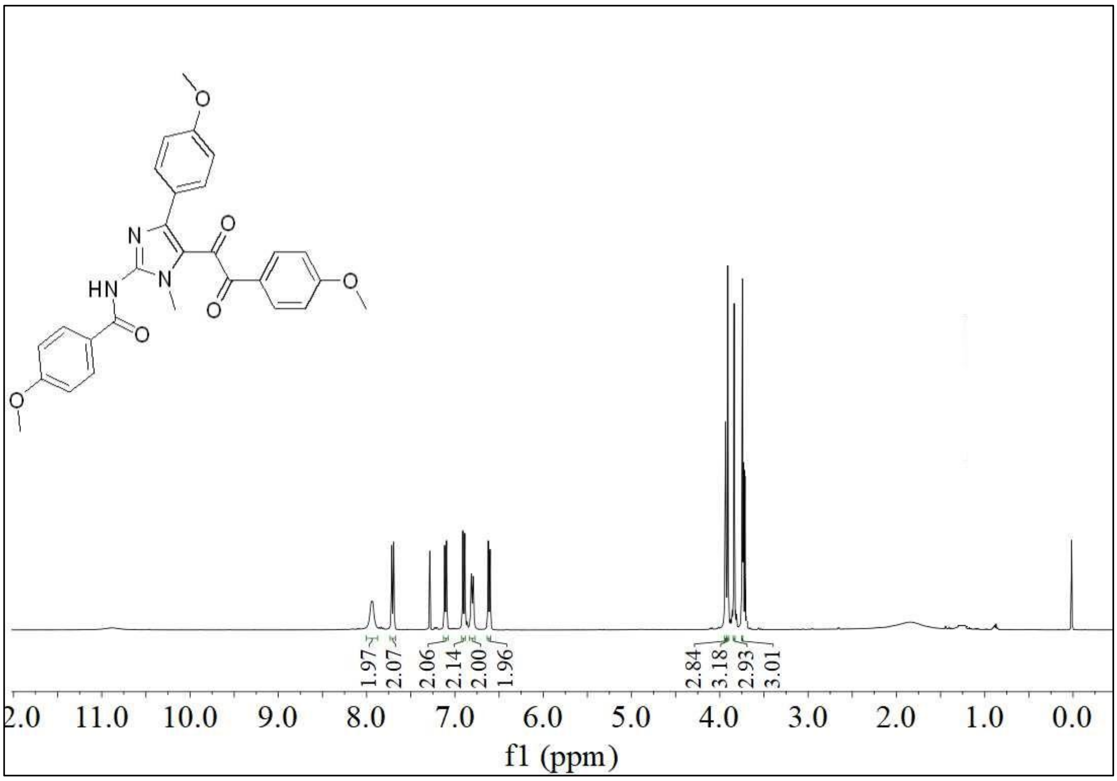

Figure S6. ^{1}H NMR Spectrum of **A$_3$** in $CDCl_3$

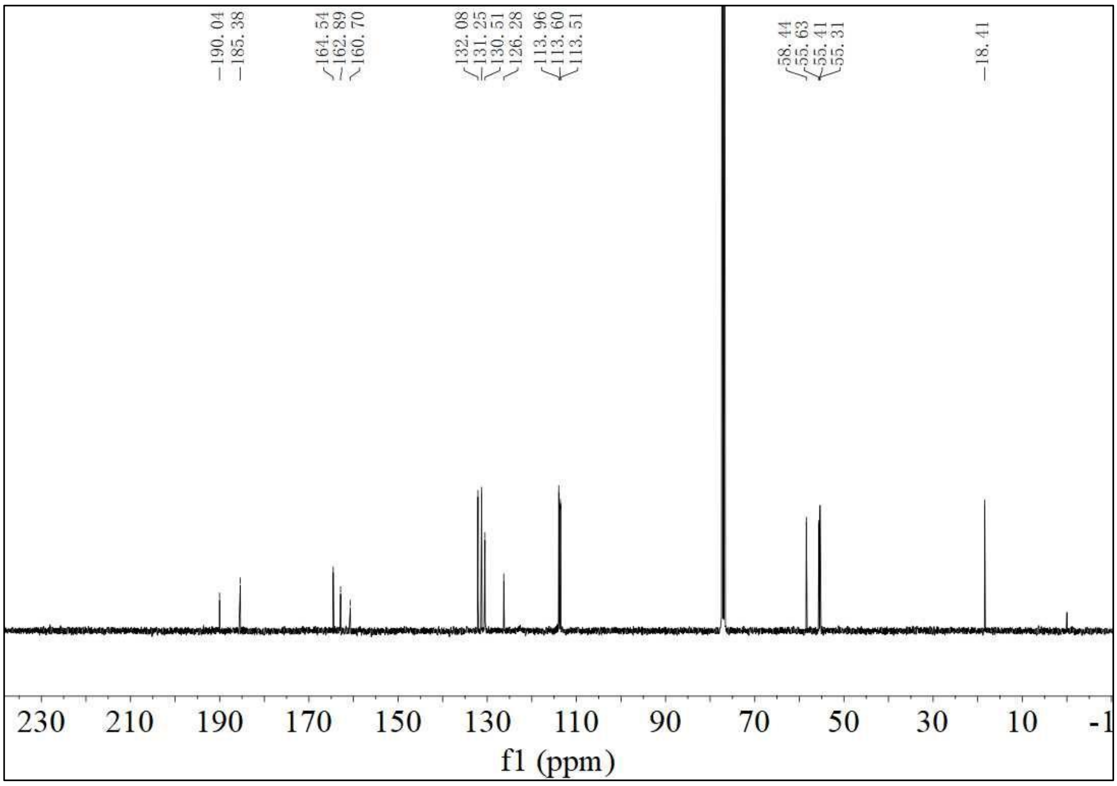

Figure S7. ^{13}C NMR Spectrum of **A$_3$** in $CDCl_3$

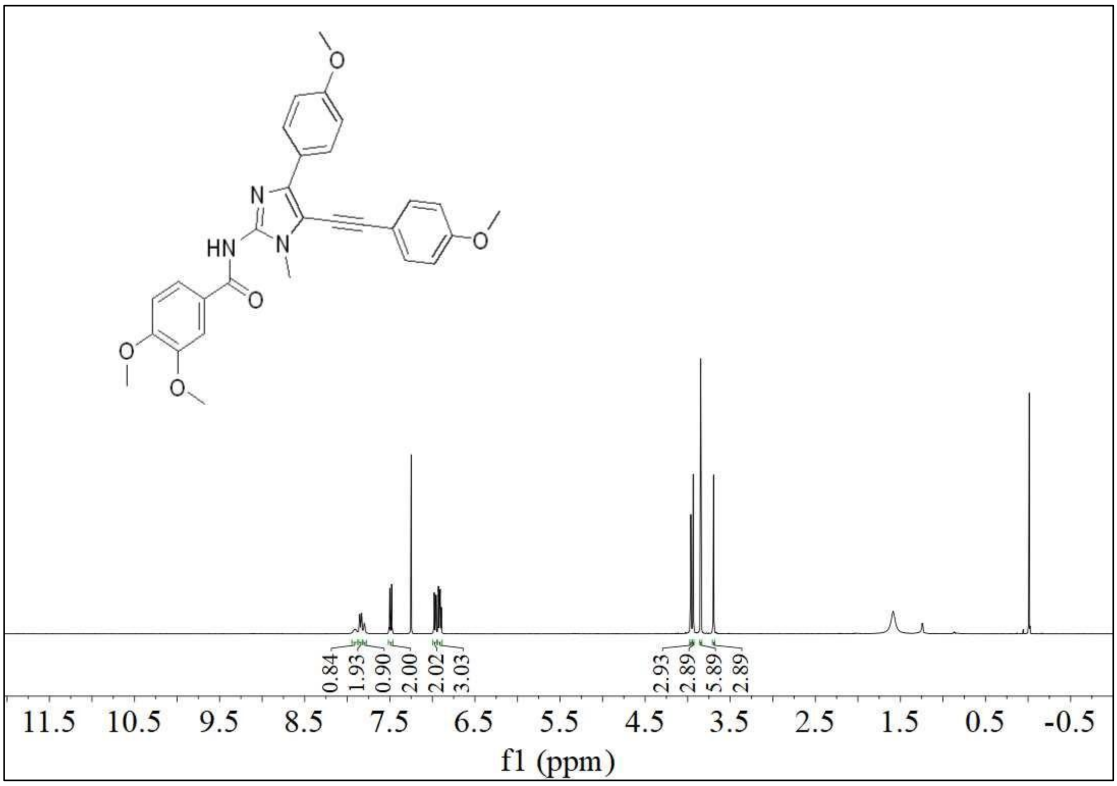

Figure S8. ^{1}H NMR Spectrum of **B$_1$** in $CDCl_3$

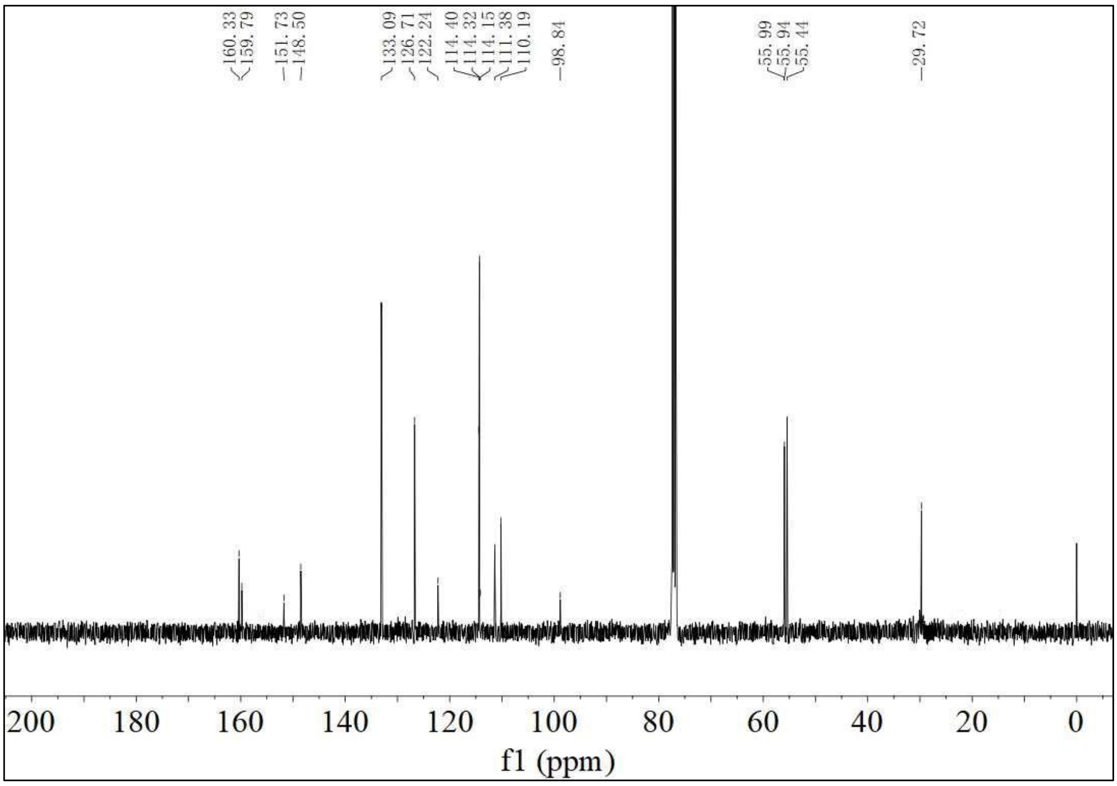

Figure S9. ^{13}C NMR Spectrum of **B$_1$** in $CDCl_3$

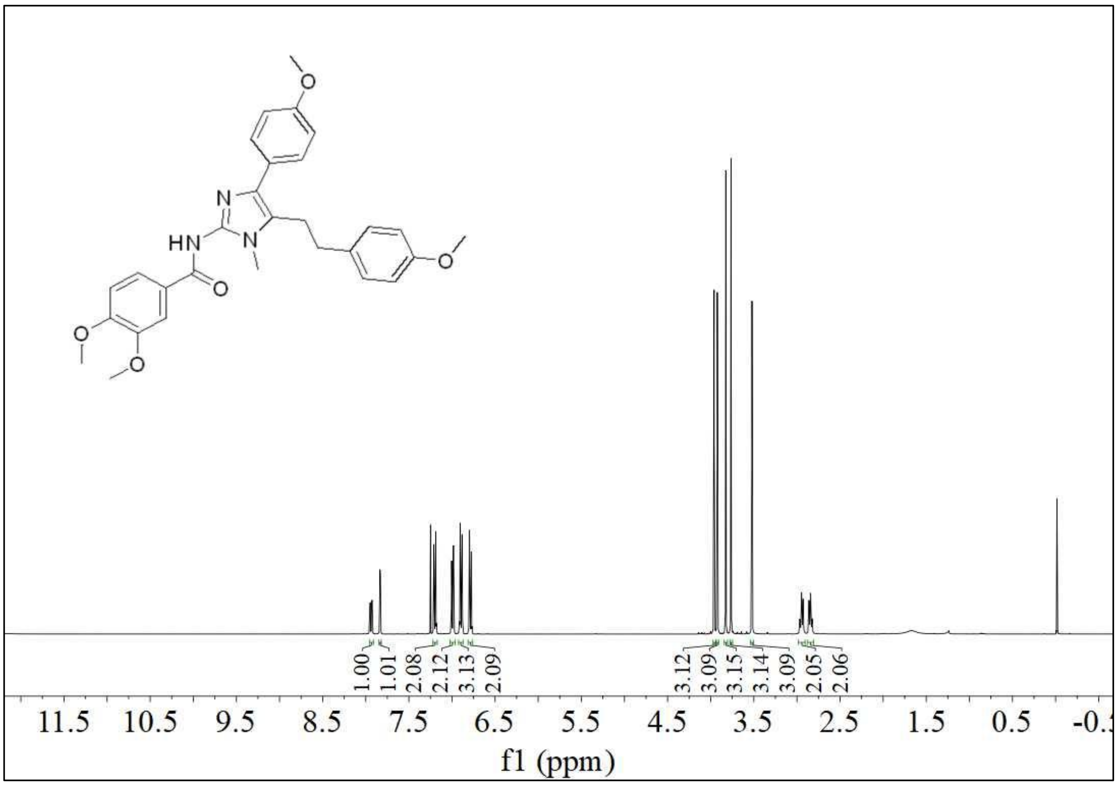

Figure S10. ^{1}H NMR Spectrum of **B_2** in $CDCl_3$

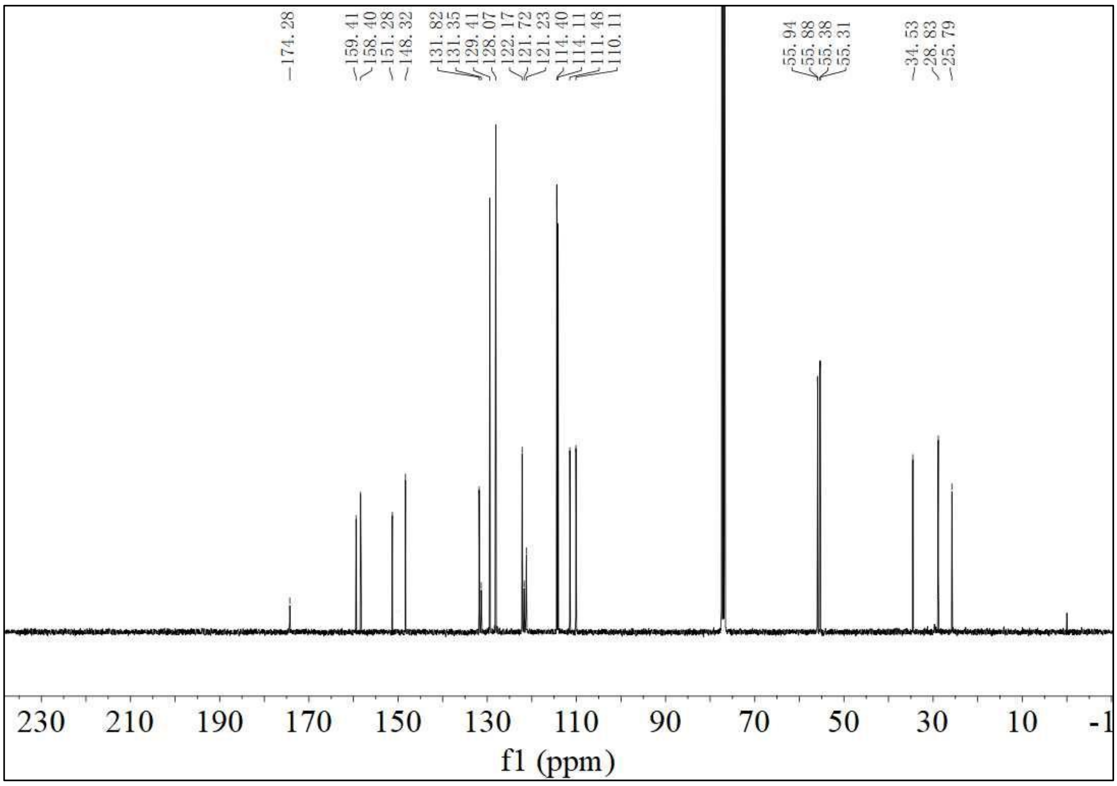

Figure S11. ^{13}C NMR Spectrum of **B_2** in $CDCl_3$

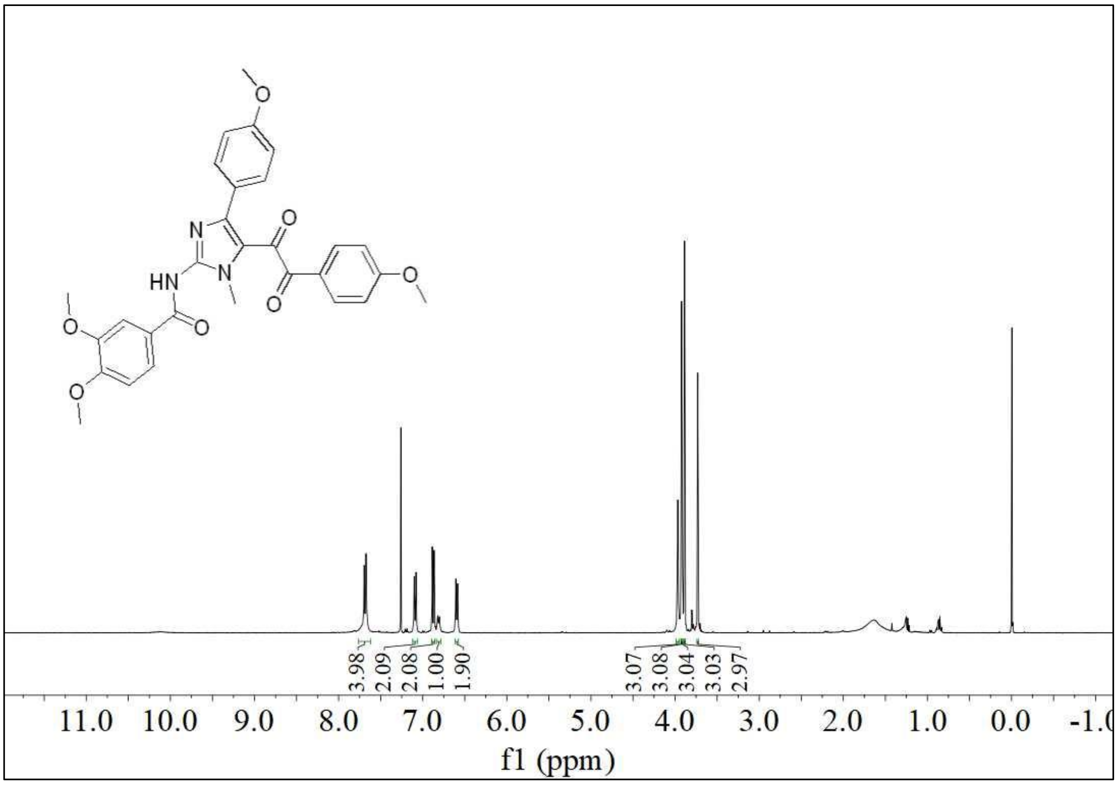

Figure S12. ^{1}H NMR Spectrum of **B_3** in $CDCl_3$

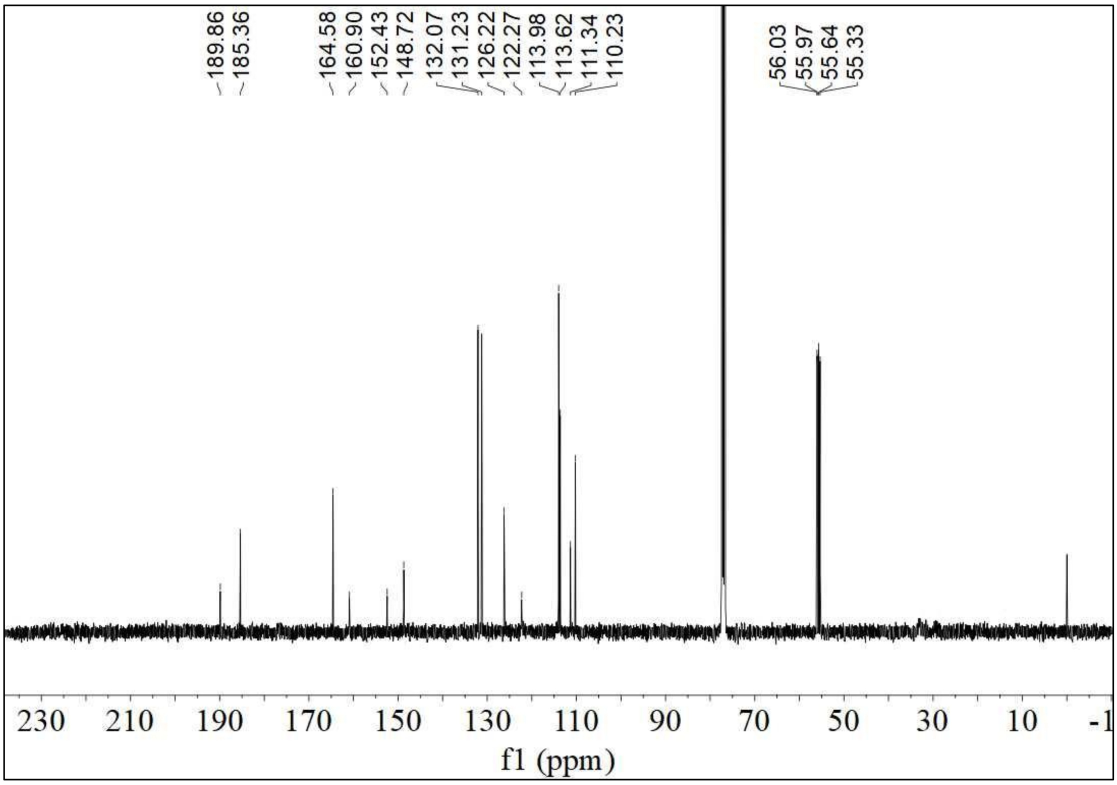

Figure S13. ^{13}C NMR Spectrum of **B_3** in $CDCl_3$

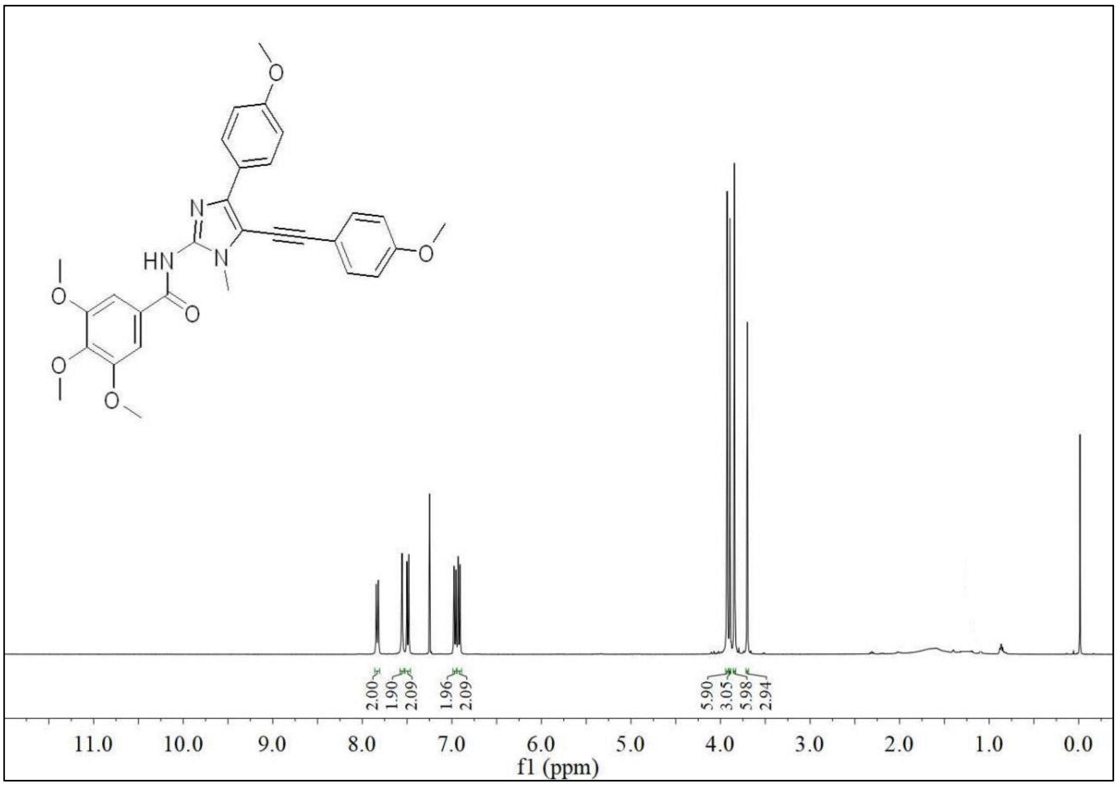

Figure S14. ^{1}H NMR Spectrum of $\mathbf{C_1}$ in $CDCl_3$

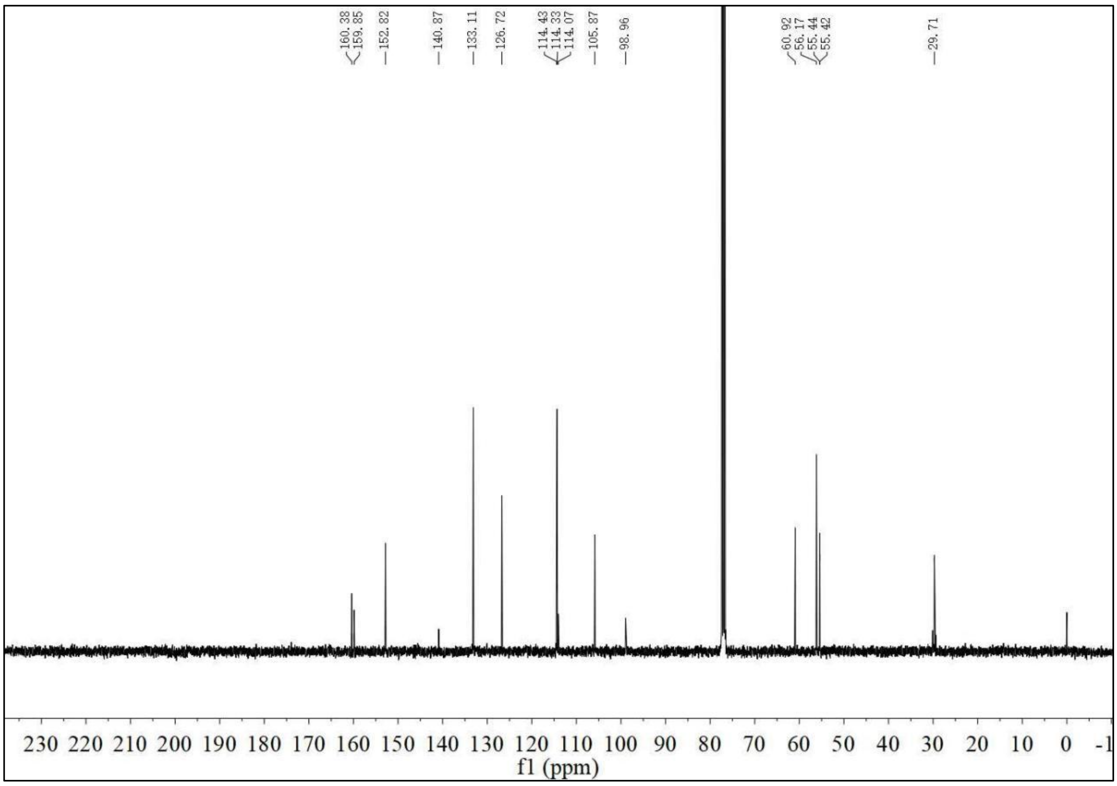

Figure S15. ^{13}C NMR Spectrum of $\mathbf{C_1}$ in $CDCl_3$

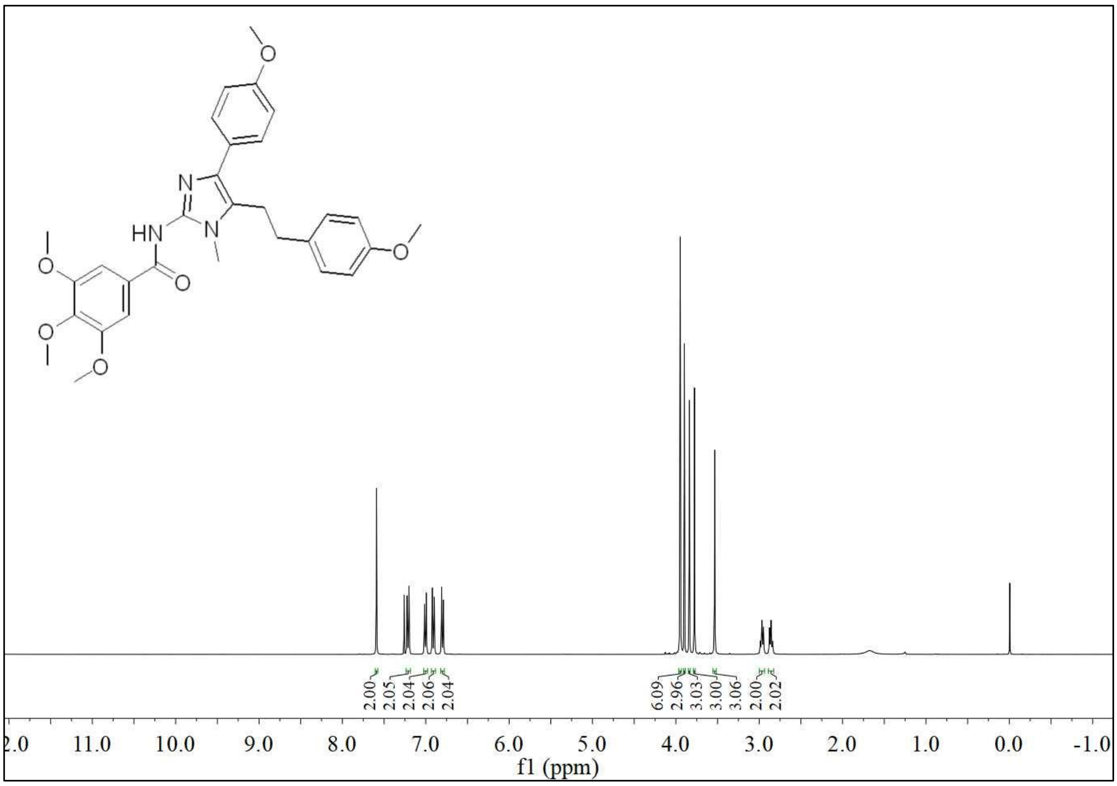

Figure S16. ^{1}H NMR Spectrum of $\mathbf{C_2}$ in $CDCl_3$

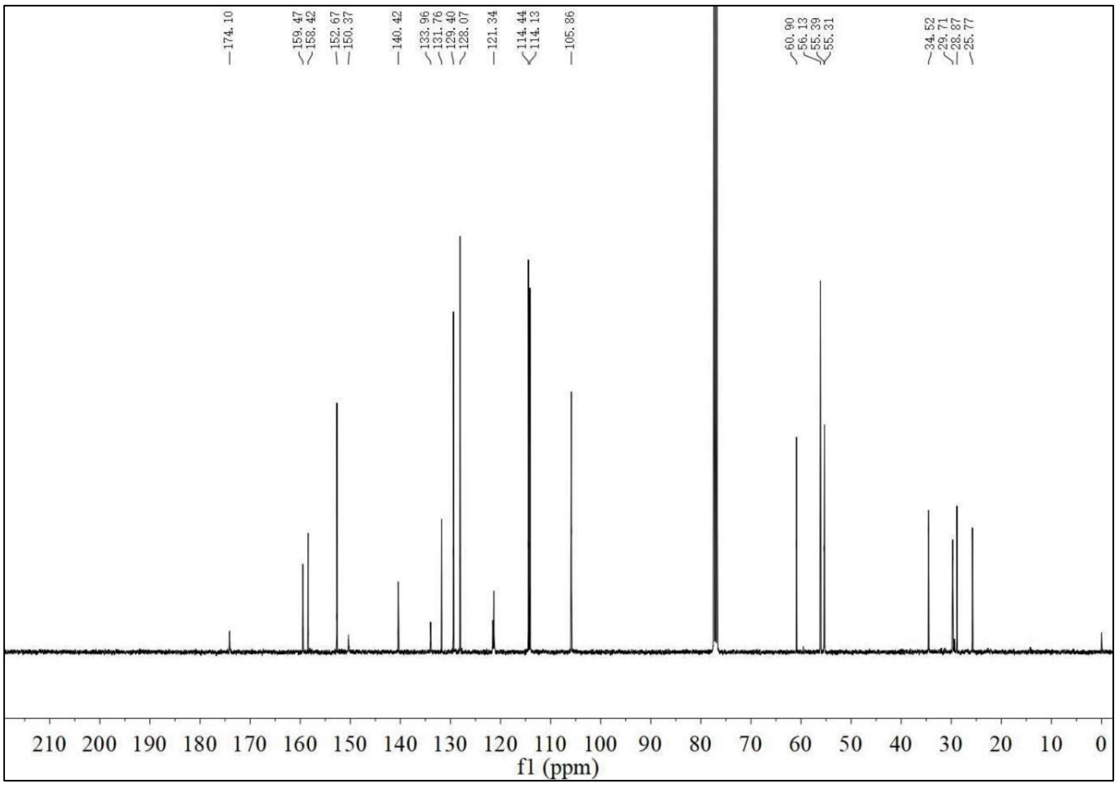

Figure S17. ^{13}C NMR Spectrum of $\mathbf{C_2}$ in $CDCl_3$

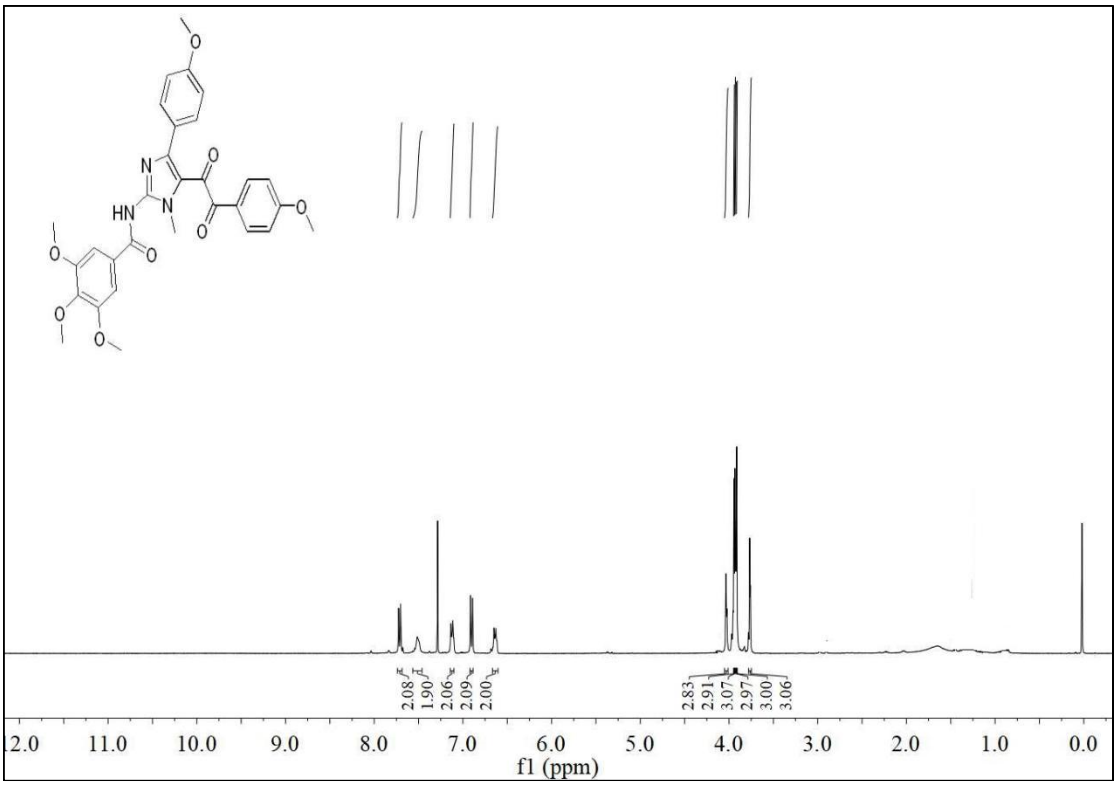

Figure S18. ^{1}H NMR Spectrum of $\mathbf{C_3}$ in $CDCl_3$

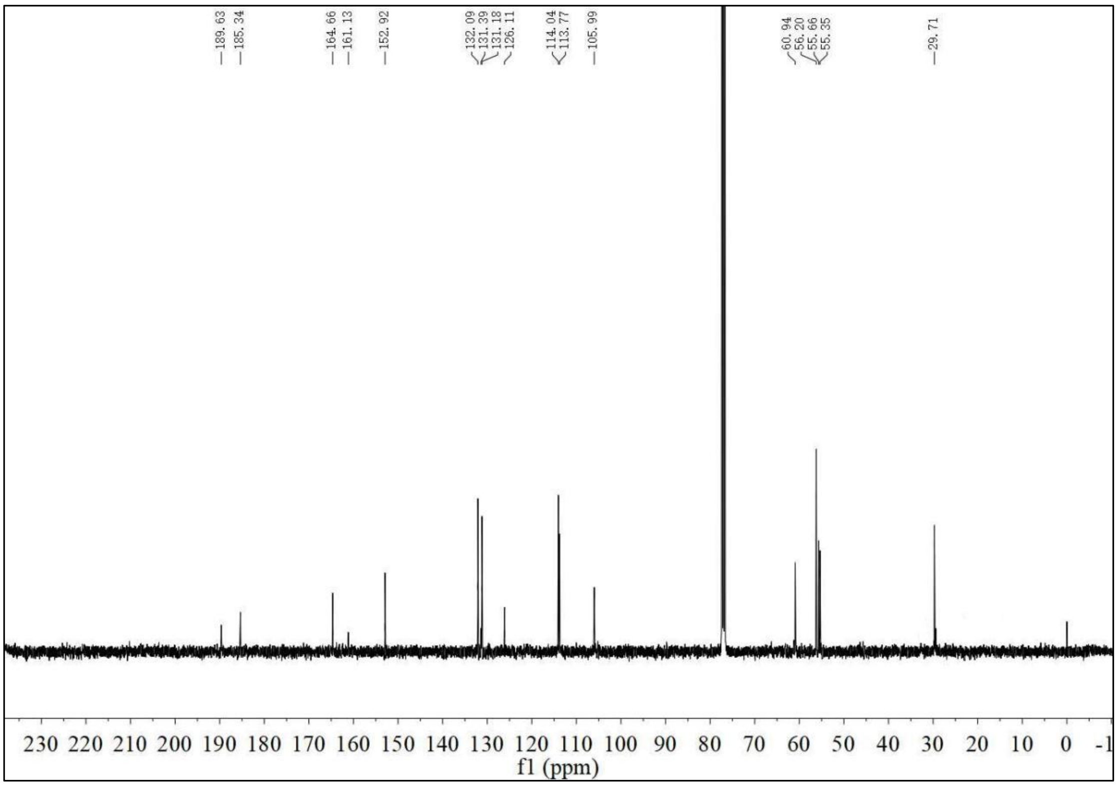

Figure S19. ^{13}C NMR Spectrum of $\mathbf{C_3}$ in $CDCl_3$

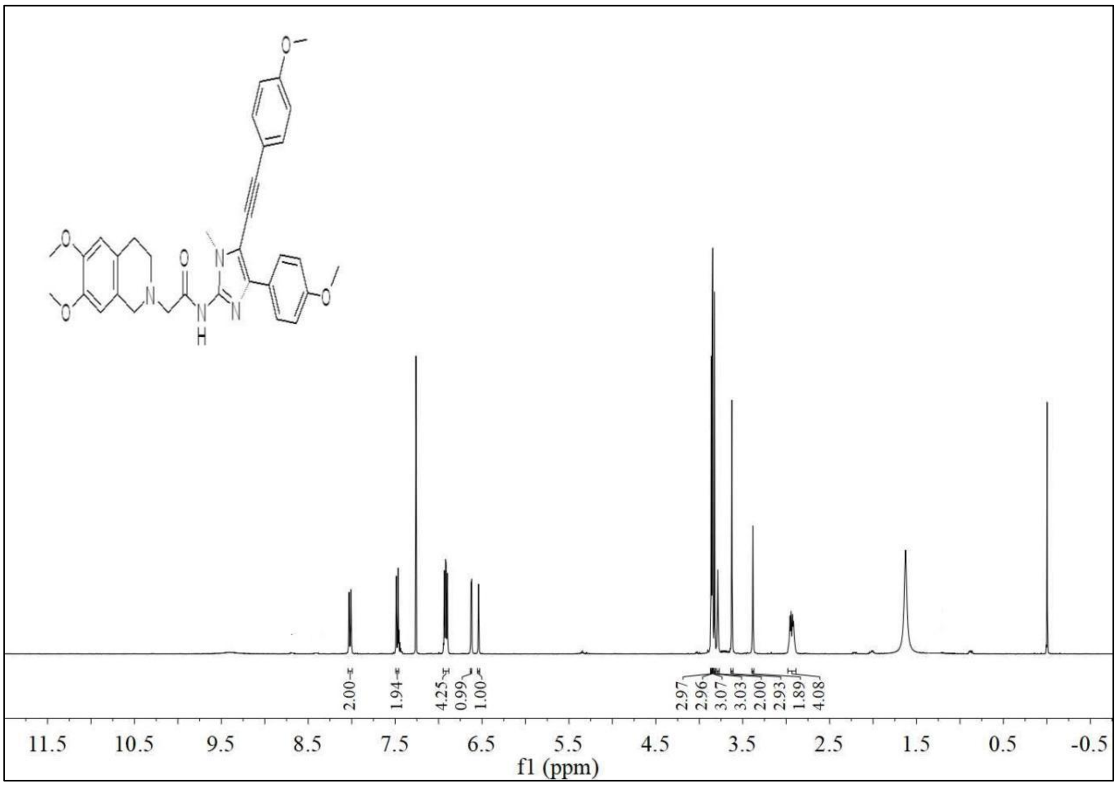

Figure S20. ^{1}H NMR Spectrum of $\mathbf{D_1}$ in $CDCl_3$

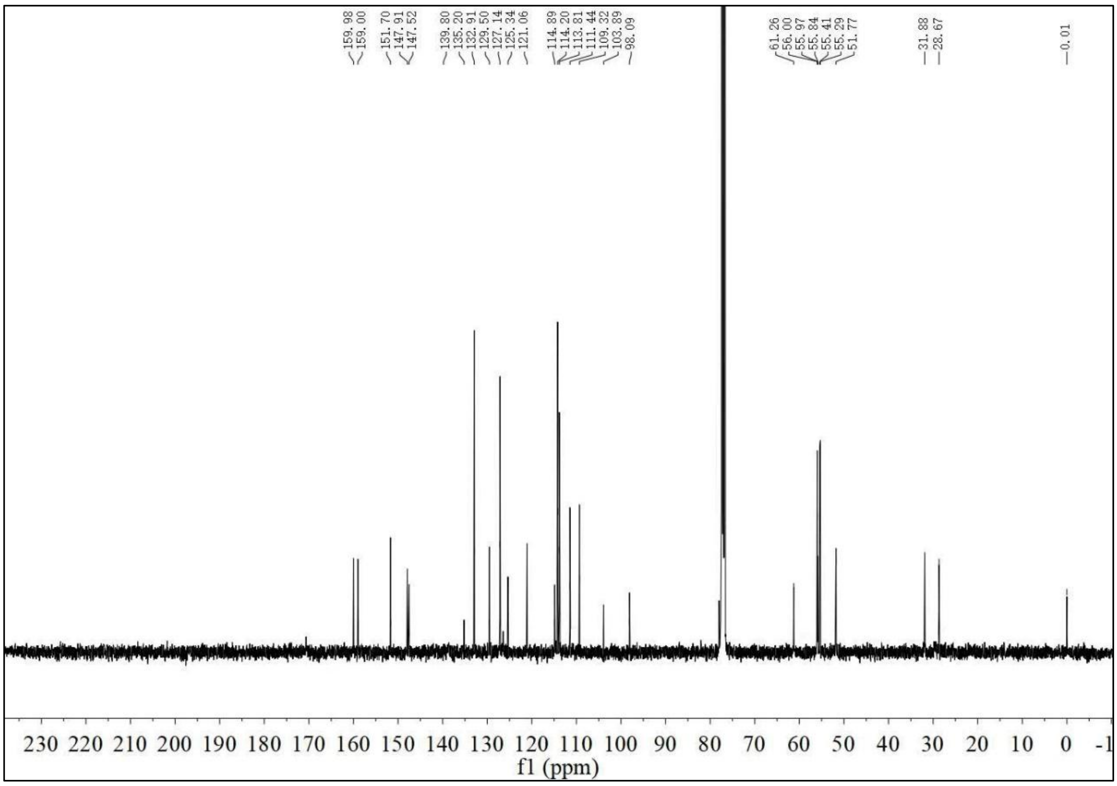

Figure S21. ^{13}C NMR Spectrum of $\mathbf{D_1}$ in $CDCl_3$

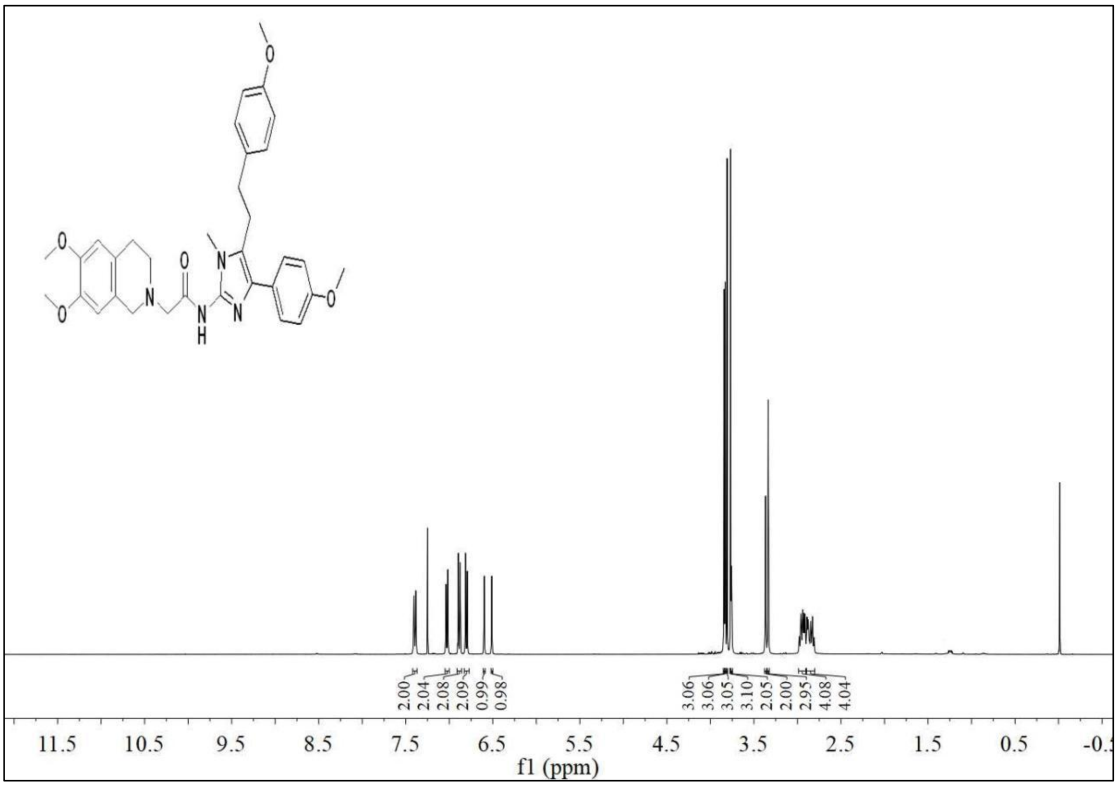

Figure S22. ^{1}H NMR Spectrum of **D$_2$** in $CDCl_3$

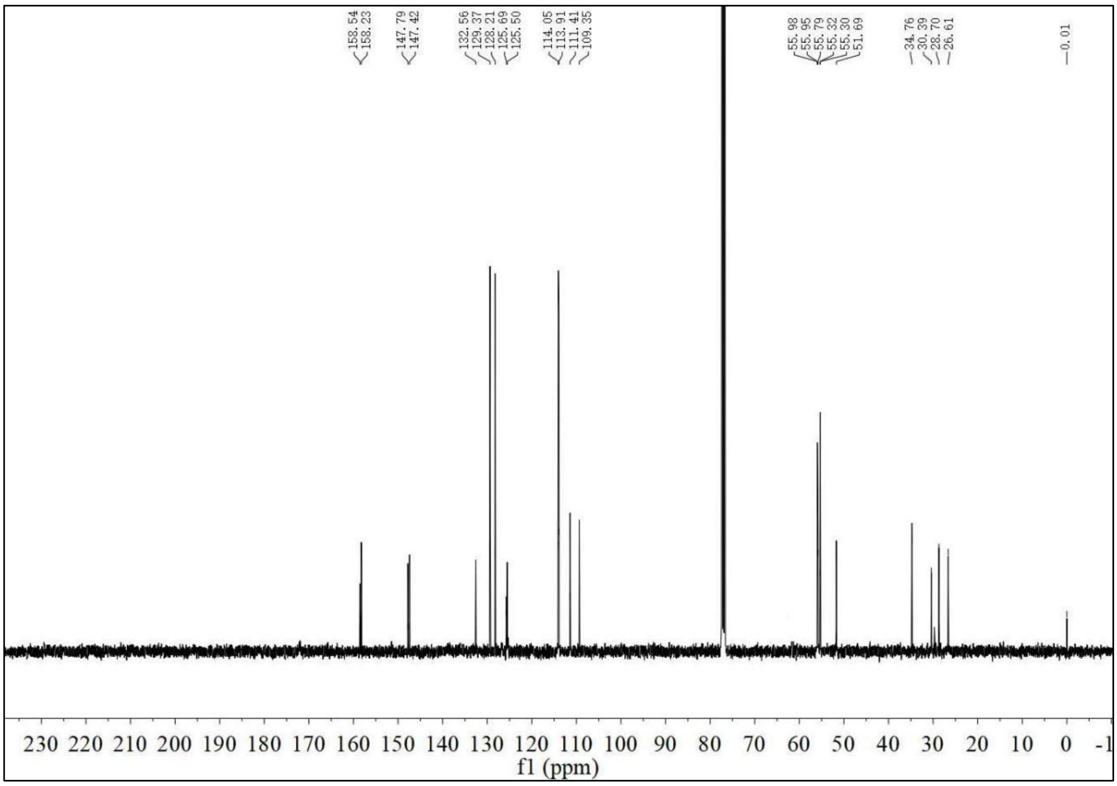

Figure S23. ^{13}C NMR Spectrum of **D$_2$** in $CDCl_3$

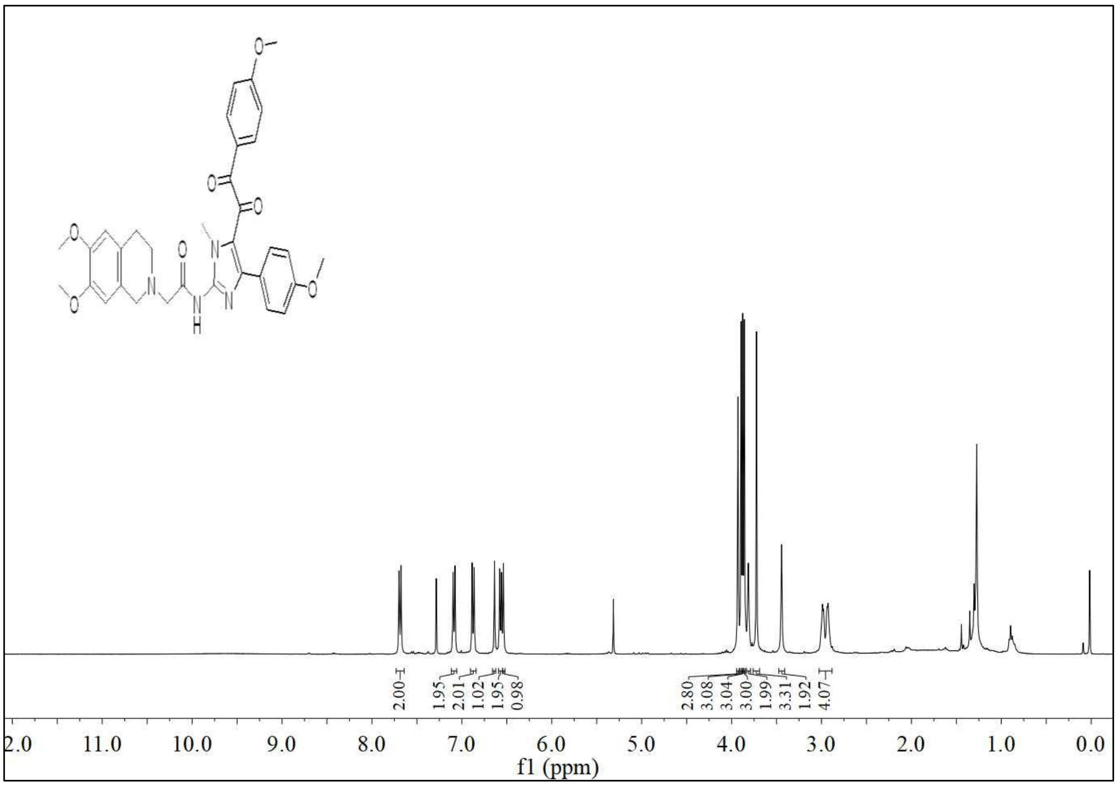

Figure S24. ^{1}H NMR Spectrum of **D3** in $CDCl_3$

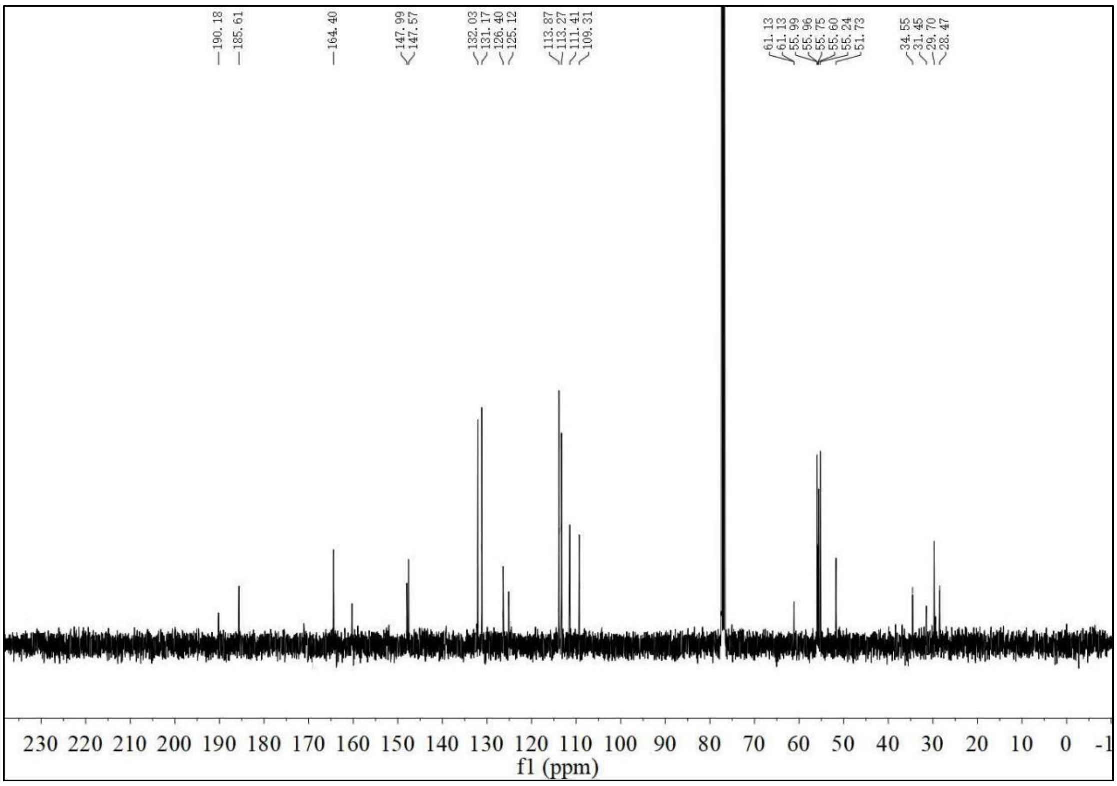

Figure S25. ^{13}C NMR Spectrum of **D3** in $CDCl_3$

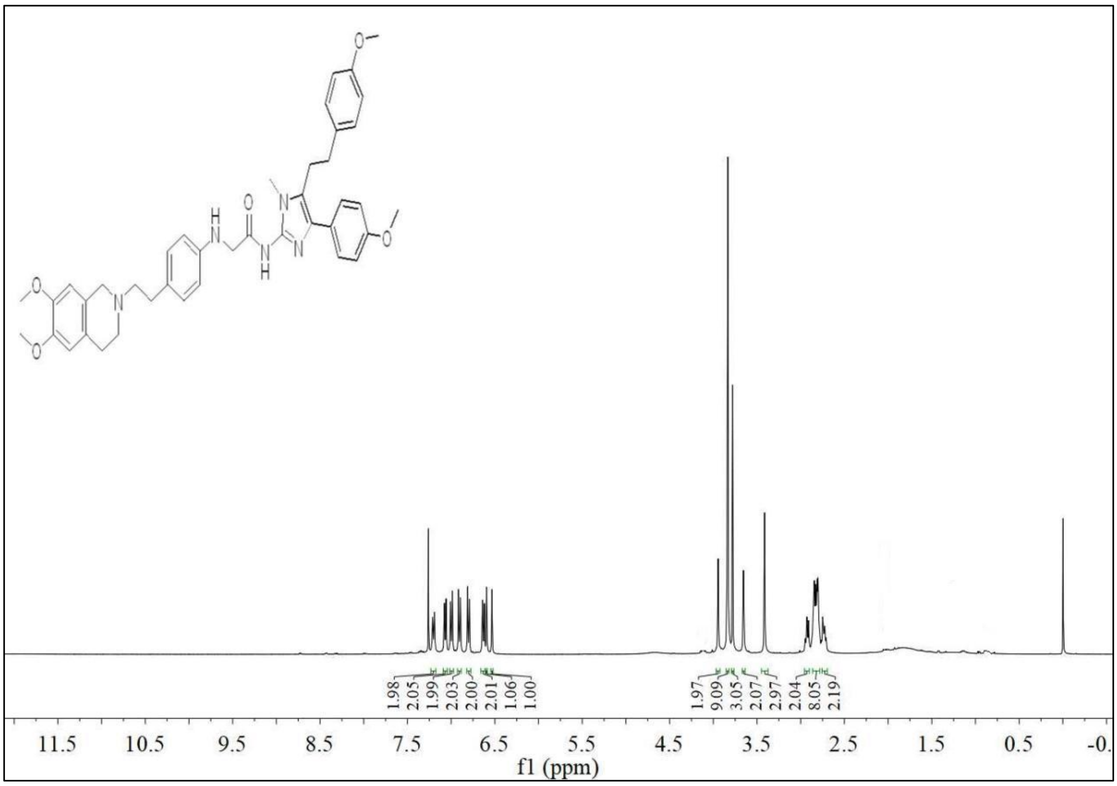

Figure S26. ^{1}H NMR Spectrum of **D_4** in $CDCl_3$

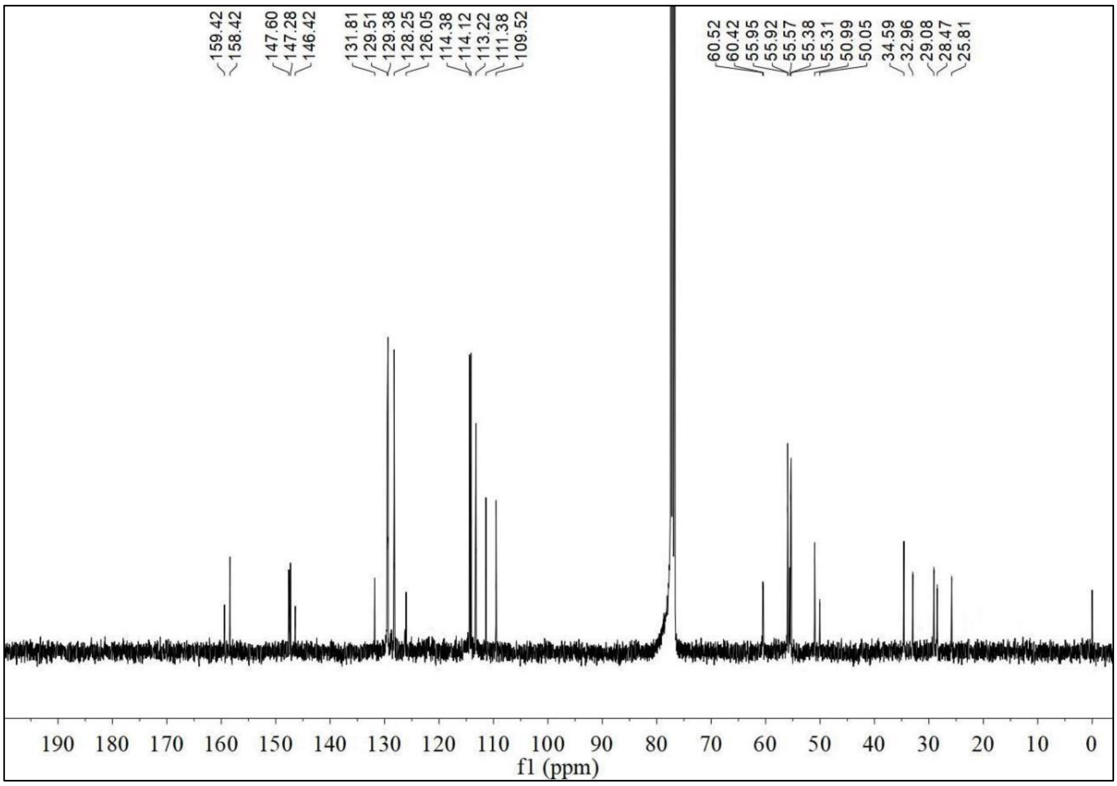

Figure S27. ^{13}C NMR Spectrum of **D_4** in $CDCl_3$

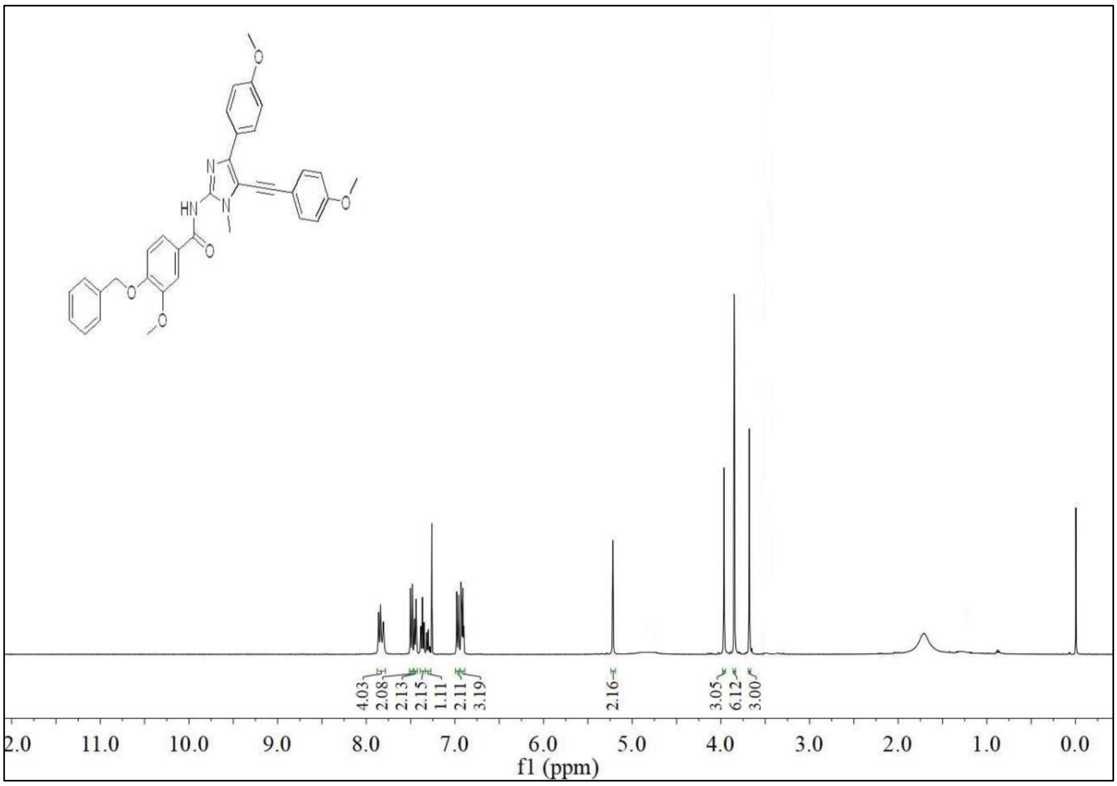

Figure S28.^{1}H NMR Spectrum of $\mathbf{E_1}$ in $CDCl_3$

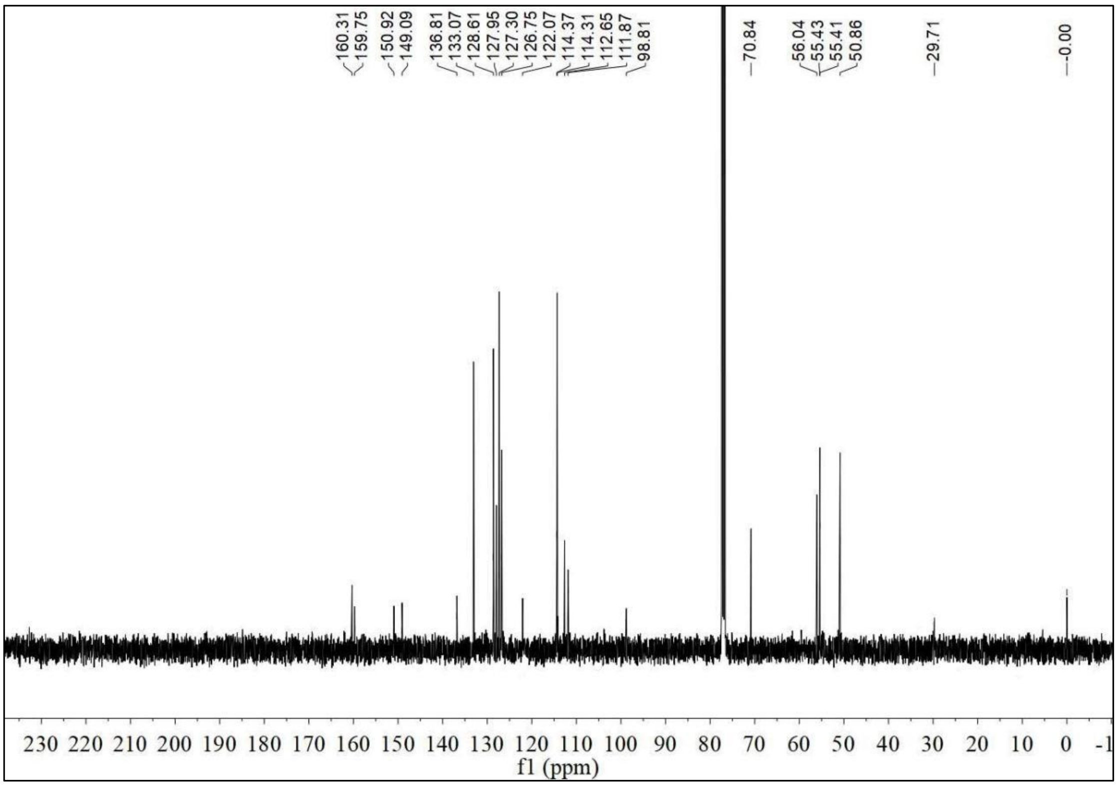

Figure S29. ^{13}C NMR Spectrum of $\mathbf{E_1}$ in $CDCl_3$

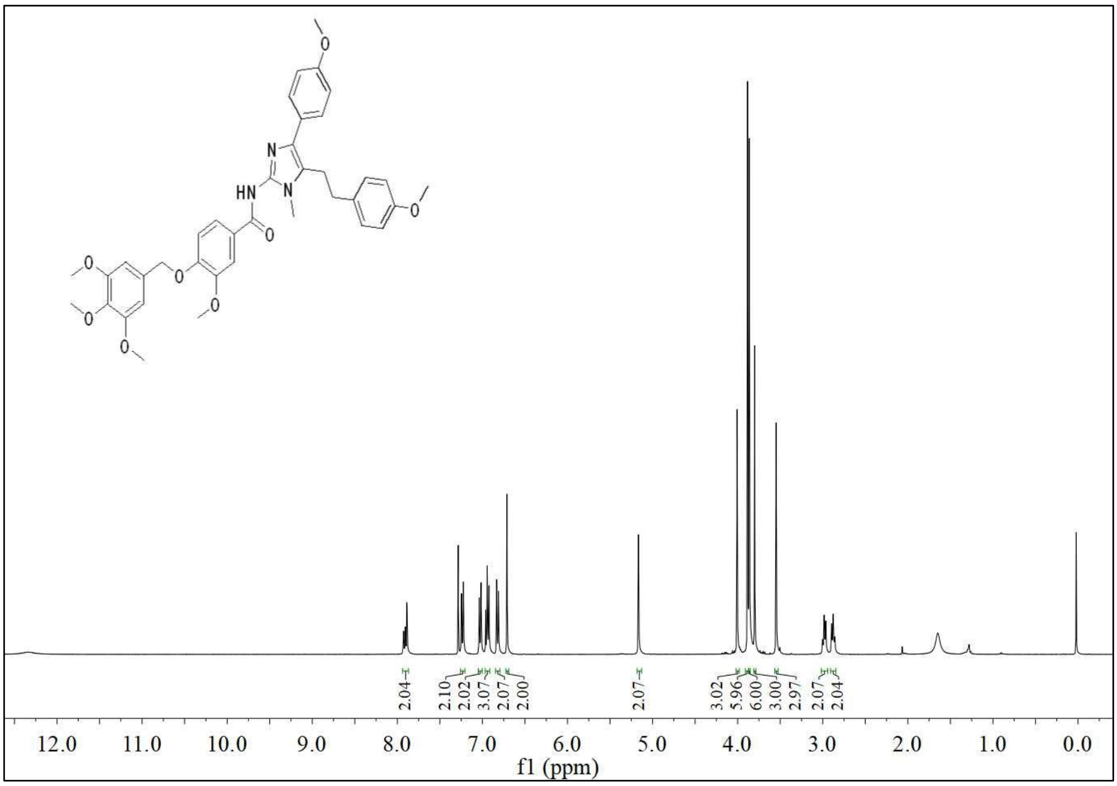

Figure S30. ^{1}H NMR Spectrum of $\mathbf{E_2}$ in $CDCl_3$

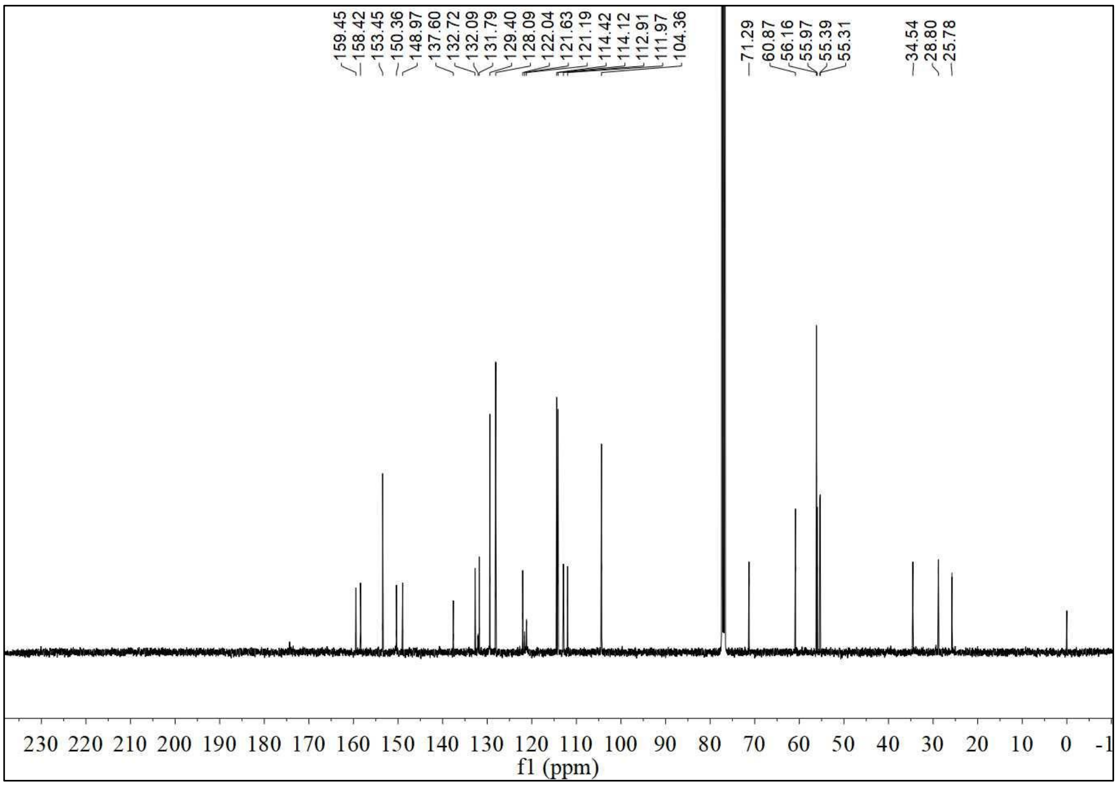

Figure S31. ^{13}C NMR Spectrum of $\mathbf{E_2}$ in $CDCl_3$

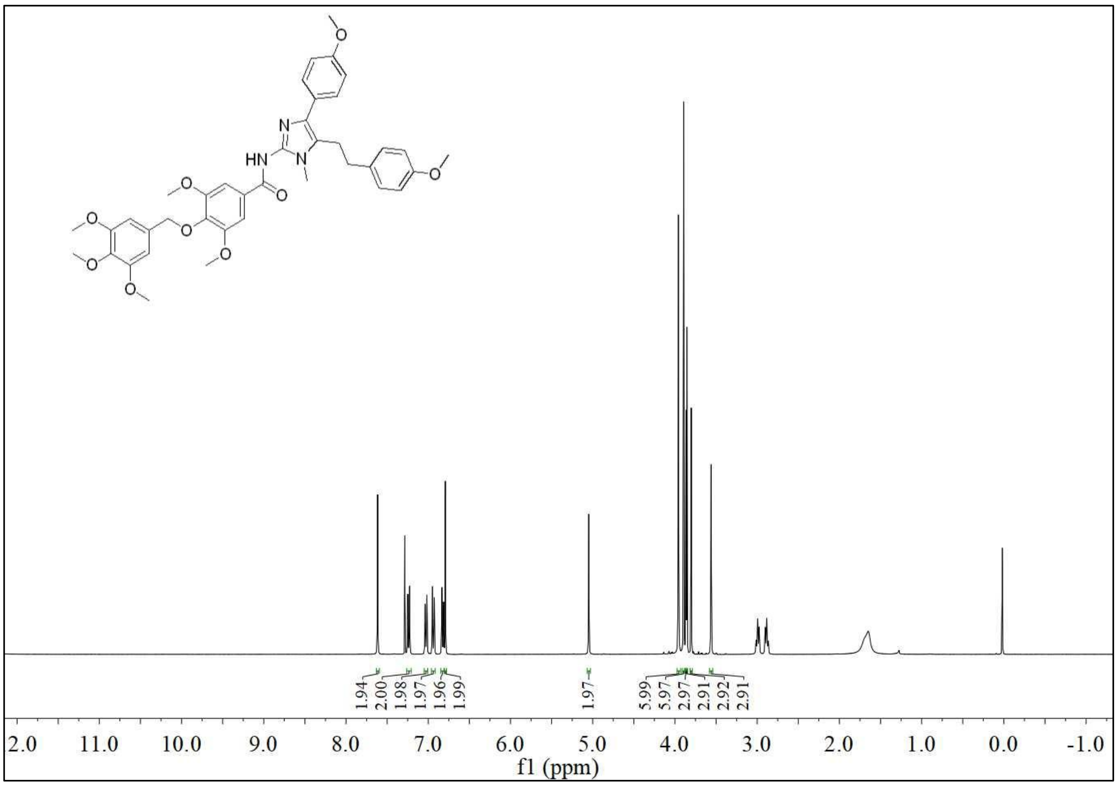

Figure S32. ^{1}H NMR Spectrum of **E_3** in $CDCl_3$

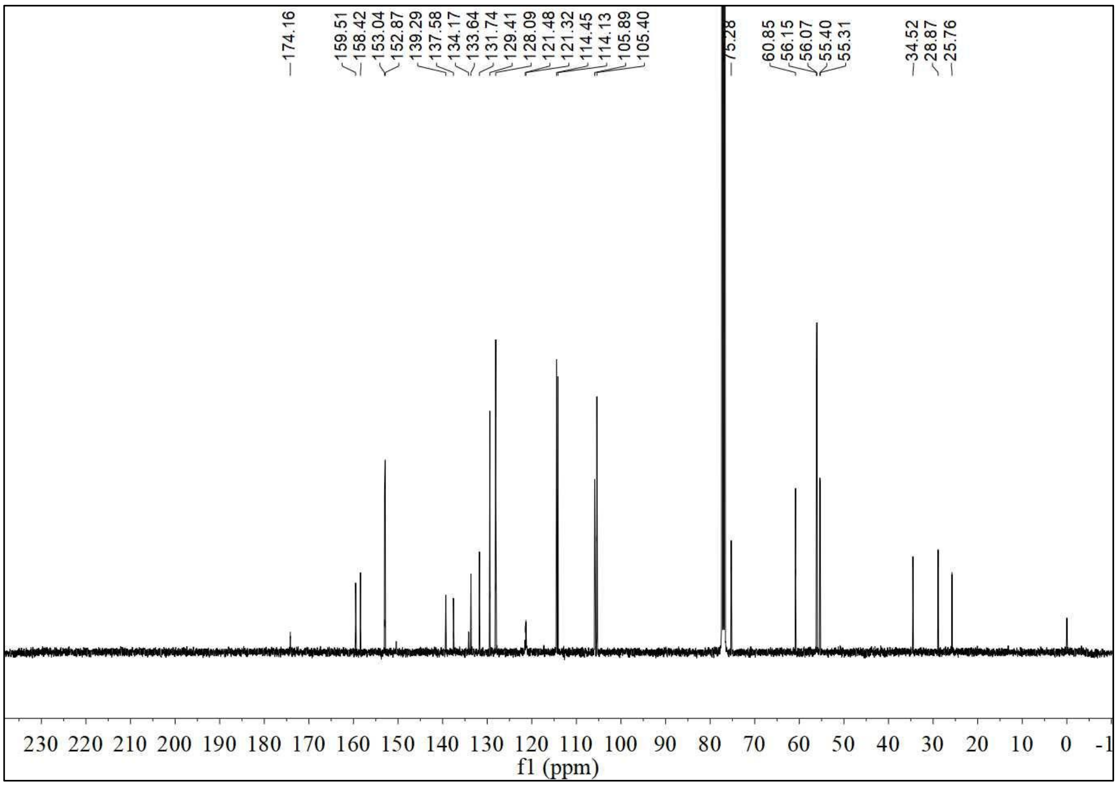

Figure S33. ^{13}C NMR Spectrum of **E_3** in $CDCl_3$

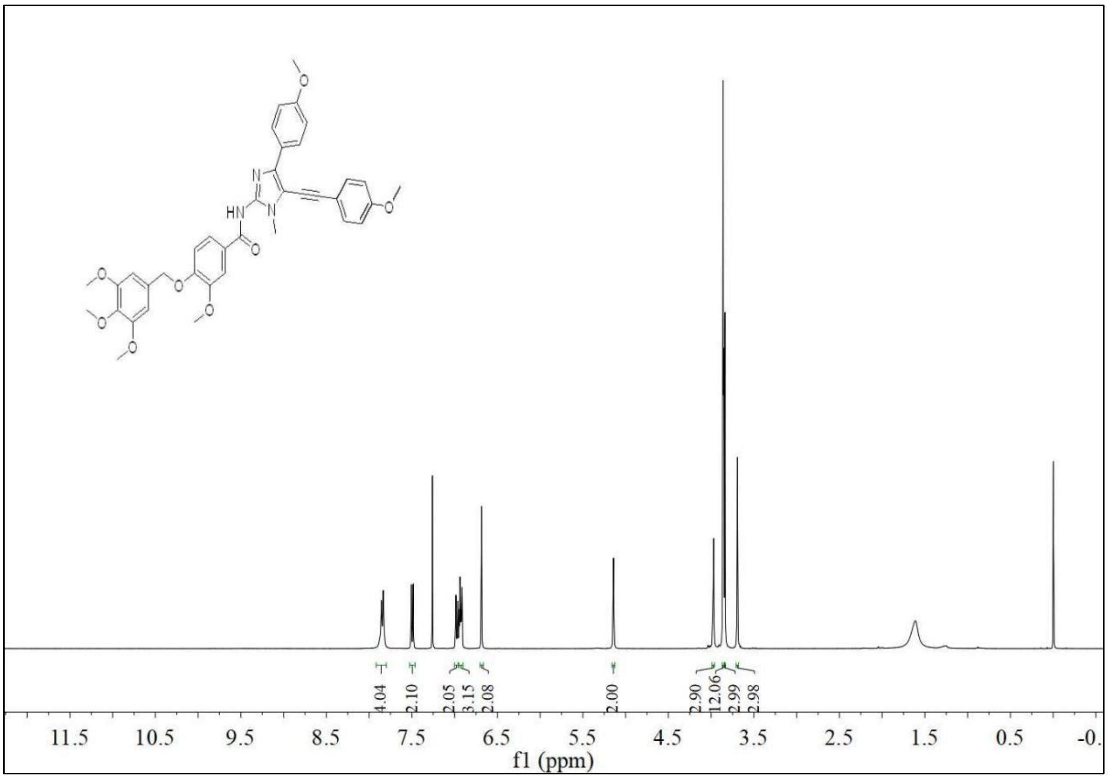

Figure S34. ^{1}H NMR Spectrum of **E$_4$** in $CDCl_3$

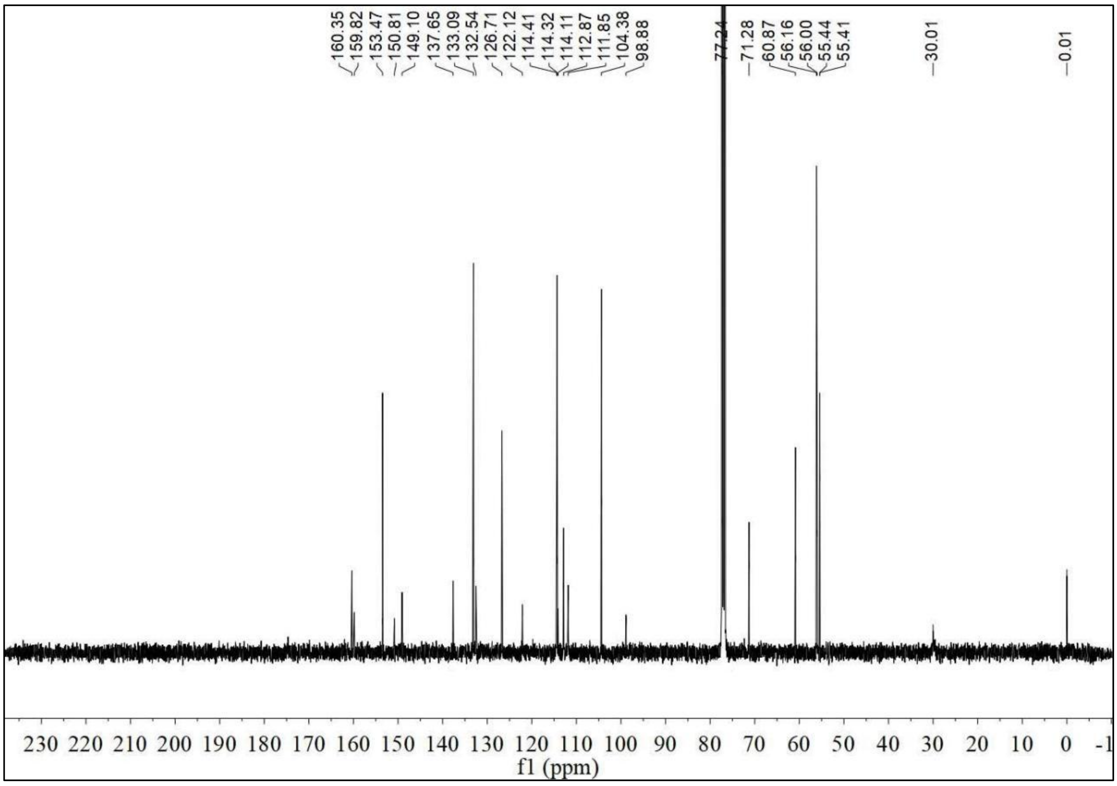

Figure S35. ^{13}C NMR Spectrum of **E$_4$** in $CDCl_3$

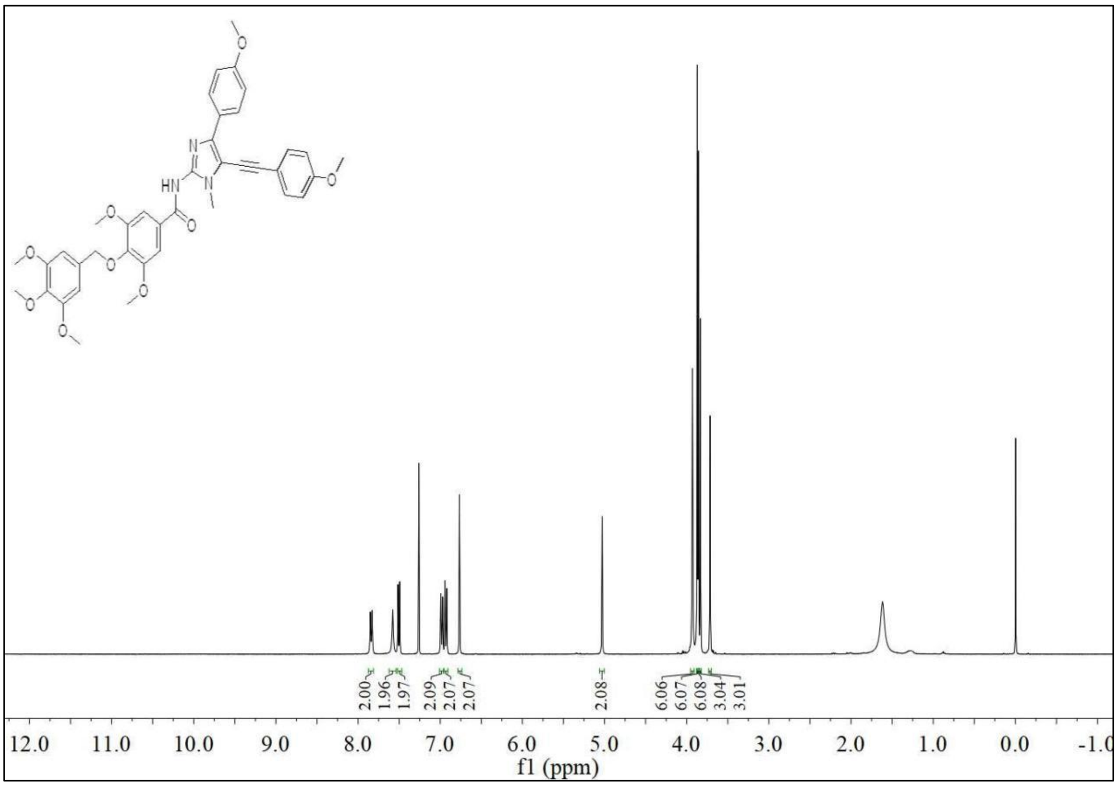

Figure S36.^{1}H NMR Spectrum of **E_5** in $CDCl_3$

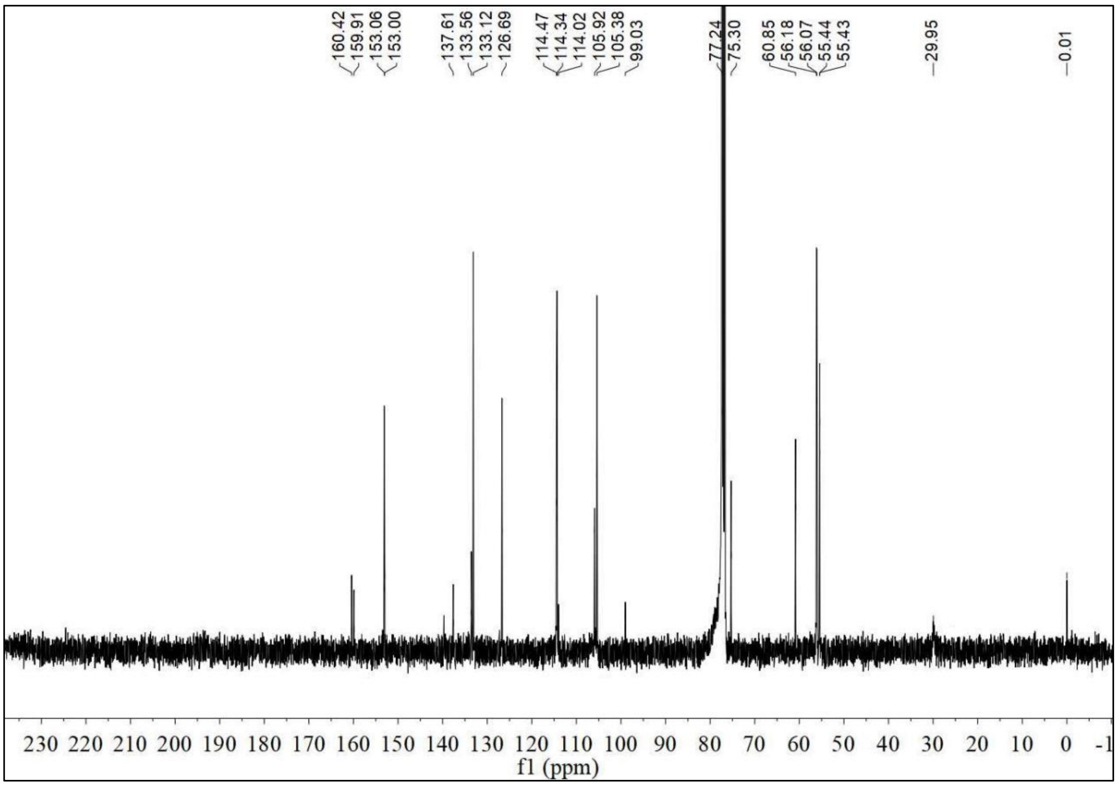

Figure S37. ^{13}C NMR Spectrum of **E_5** in $CDCl_3$

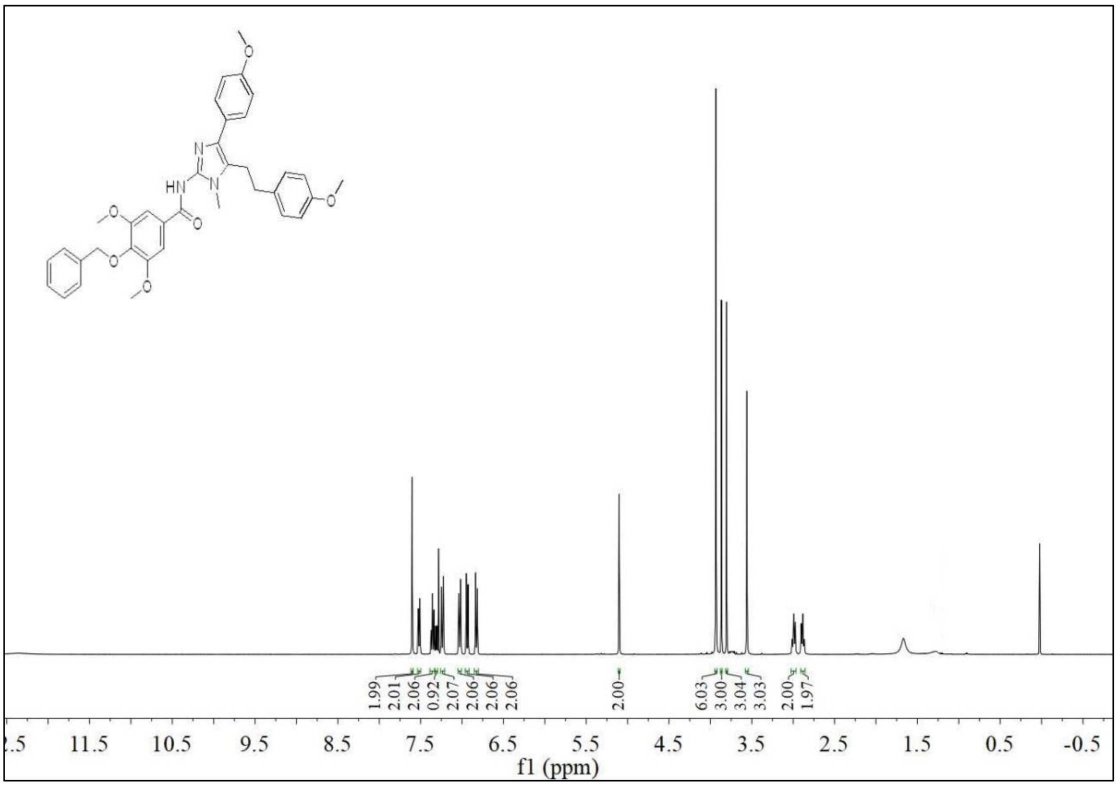

Figure S38. ^{1}H NMR Spectrum of $\mathbf{E_6}$ in $CDCl_3$

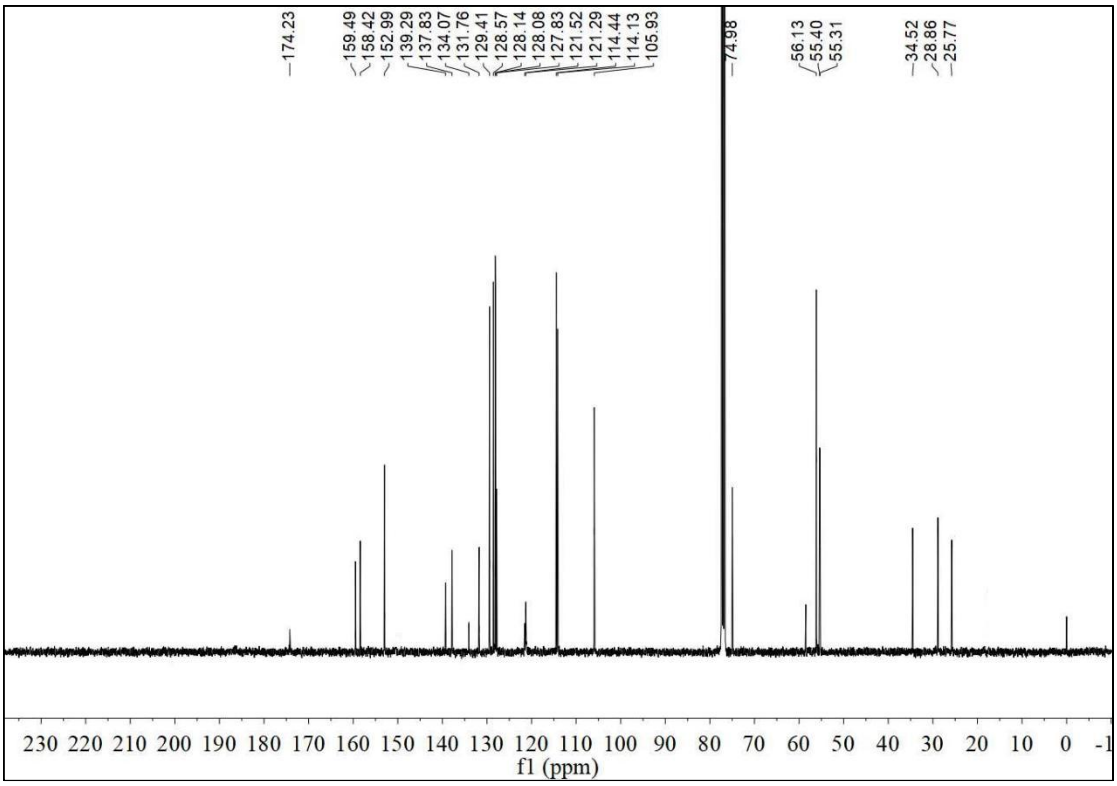

Figure S39. ^{13}C NMR Spectrum of $\mathbf{E_6}$ in $CDCl_3$
